# MSCs Suppress Macrophage Necroptosis and Foster Liver Regeneration by Modulating SP1/SK1 Axis in Treating Acute Severe Autoimmune Hepatitis

**DOI:** 10.1002/advs.202408974

**Published:** 2025-02-03

**Authors:** Ran An, Zhengyi Zhu, Yuyan Chen, Wenxian Guan, Jinglin Wang, Haozhen Ren

**Affiliations:** ^1^ Division of Hepatobiliary and Transplantation Surgery Department of General Surgery Nanjing Drum Tower Hospital the Affiliated Hospital of Medical School Nanjing University Nanjing 210008 China

**Keywords:** autoimmune hepatitis, liver regeneration, macrophage, mesenchymal stem cells, necroptosis

## Abstract

Acute severe autoimmune hepatitis (AS‐AIH) is characterized by rapid progression and poor prognosis, with a current lack of effective targeted treatments. Stem cell therapy has demonstrated significant therapeutic promise across various autoimmune diseases. However, the intricate pathogenesis of AS‐AIH has hindered the widespread utilization of mesenchymal stem cells (MSCs) in this domain. Herein, it is demonstrated that necroptosis, as the primary mode of cell death in AIH, is crucial in causing AS‐AIH. Inflammatory macrophages are the primary cell population involved in necroptosis. Inhibition of the specificity protein 1/sphingosine kinase 1/sphingosine‐1‐phosphate (SP1/SK1/S1P) axis is responsible for this phenomenon, leading to excessive activation of the intrahepatic immune system and aggravating liver damage. Furthermore, the S1P/S1PR2/YAP axis is the key pathway in initiating liver regeneration during AS‐AIH. S1P synthesized by hepatocytes is the primary source, and this process is also regulated by the SP1/SK1 axis. MSCs promote S1P synthesis by macrophages through the delivery of SP1, which inhibits necroptosis and synergistically enhances liver regeneration. In addition, MSCs also promote S1P synthesis in hepatocytes through the same mechanism, further aiding liver regeneration. These findings unveil the core pathogenesis of AS‐AIH and provide a theoretical foundation for using MSCs as a potential targeted therapeutic modality.

## Graphical abstract

In AS‐AIH, the SP1/SK1/S1P axis and S1P/S1PR2/YAP pathway are central to disease progression and liver regeneration. MSCs can enhance liver repair by promoting S1P synthesis, then inhibiting necroptosis, offering a potential therapeutic strategy for AS‐AIH.

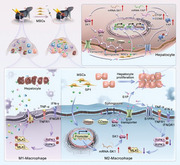

## 1 Introduction

Acute severe autoimmune hepatitis (AS‐AIH) was defined as acute severe hepatitis occurring in individuals with preexisting AIH or in the absence of any other causative factors leading to liver injury.^[^ ^1^ ^]^ The pathogenesis of AIH is influenced by a multitude of factors, with genetic predisposition and environmental determinants being pivotal.^[^ ^2^ ^]^ Despite AIH's hallmark of indolent onset and gradual progression, the prognosis is often grim. Approximately one‐fourth of patients exhibit signs of cirrhosis at diagnosis, with an inherent risk of advancing to hepatic failure.^[^ ^3^ ^]^ However, a subset of studies suggests that 50–60% of patients, particularly younger demographics, present with an acute onset.^[^ ^4^, ^5^ ^]^ AS‐AIH represents a distinct variant of acute AIH, characterized by an abrupt onset and swift deterioration of clinical signs. Given that conventional AIH treatments, including glucocorticoids and immunosuppressants, are frequently ineffective in AS‐AIH, over half of these patients may progress to acute liver failure (ALF), which is associated with a significantly elevated mortality rate.^[^ ^1^, ^6^ ^]^ Consequently, there is an exigent need for efficacious therapeutic interventions to mitigate the severity of AS‐AIH and enhance patient prognoses. As is known to all, the imbalance of the intrahepatic immune microenvironment serves as a core mechanism leading to ALF.^[^ ^7^, ^8^ ^]^ The liver is abundant in various immune cells, which collaborate to eliminate harmful substances and maintain intrahepatic immune homeostasis. Excessive activation or suppression of the intrahepatic immune system may lead to exacerbated liver injury or hindered liver regeneration. As an immune‐mediated acute liver injury, it is crucial to clarify the state of intrahepatic immune microenvironment in the progression of AS‐AIH.

Cell death has consistently been a prominent topic in research on organ injury. Studies have shown that necroptosis is the predominant mode of cell death in AIH.^[^ ^9^, ^10^, ^11^, ^12^ ^]^ As a type of programmed cell death, necroptosis was initially discovered to be triggered by tumor necrosis factor‐alpha (TNF‐α). The central mechanism of this process involves the phosphorylation of mixed lineage kinase domain‐like (MLKL), which is regulated by receptor‐interacting serine/threonine‐protein kinase 1/3 (RIPK1/3). Eventually, the cell membrane ruptures, leading to the release of damage‐associated molecular patterns.^[^ ^13^ ^]^ This may lead to further activation of the immune system and the release of large amounts of proinflammatory factors. Hence, it is imperative to investigate whether necroptosis is involved in the development of AS‐AIH, identify the major cell populations responding to necroptosis, and tailor treatment strategies accordingly.

Diagnosing AS‐AIH is challenging, as some patients may even lack typical AIH features such as abnormal autoantibodies.^[^ ^14^ ^]^ This leads to delayed treatment or suboptimal response to classic AIH treatments, ultimately resulting in ALF. Mesenchymal stem cells (MSCs) have been gradually applied to various diseases, particularly autoimmune disorders, due to their capability to differentiate into diverse cell types, suppress immune responses, facilitate tissue repair, and impede cell death.^[^ ^15^, ^16^ ^]^ However, the core pathogenesis of AS‐AIH and the suitability of MSCs for treating this disease remain uncertain.

In this study, we elucidated the core pathogenesis of AS‐AIH and investigated its impact on the intrahepatic immune microenvironment. Building on this, we examined the therapeutic effect and specific mechanism of MSCs in treating AS‐AIH.

## 2 Results

### 2.1 Macrophage Necroptosis Serves as a Pivotal Factor Contributing to the Onset of AS‐AIH

**Figure 1:**
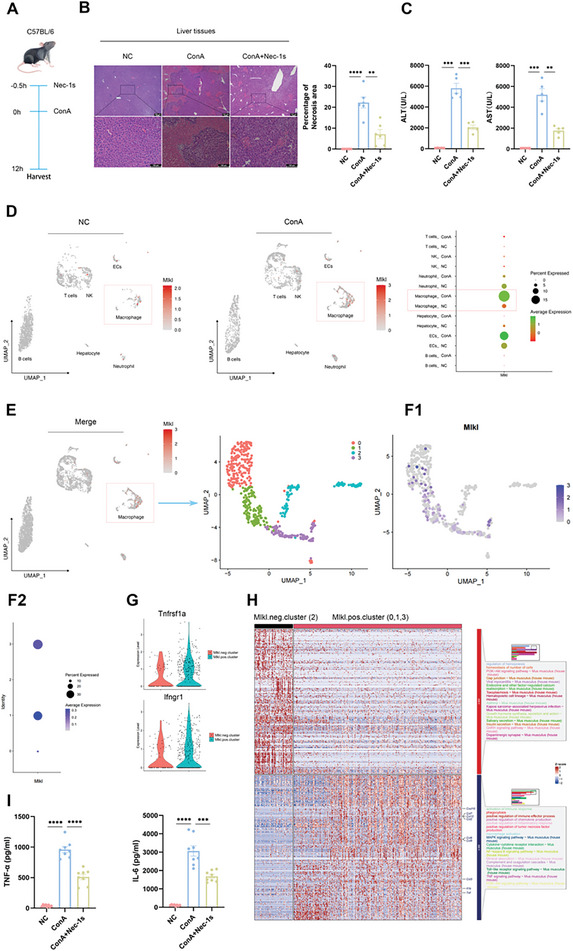
Necroptosis of macrophages serves as a pivotal factor contributing to the onset of AS‐AIH. A) Schematic of the setup for inducing AS‐AIH and treatment model in C57/BL6 mice. B) Representative H&E stained images showed the histological morphology of liver tissues (scale bars, 100 µm) (*n* = 6). C) The levels of ALT and AST in serum were detected by ELISA (*n* = 5). D) Expression levels of *Mlkl* for each cell cluster in two groups of mouse liver. E) UMAP visualization of intrahepatic macrophage subsets, annotated and colored by clustering. F) UMAP visualization of *Mlkl* expression in intrahepatic macrophages. G) Expression of *Tnfrsf1a* and *Ifngr1* in intrahepatic macrophages. H) Enrichment analysis of top 300 up‐regulated genes in *Mlkl* positive and negative clusters. I) The expressions of TNF‐α and IL‐6 in serum were tested by ELISA (*n* = 8). Data are presented as means ± SEM, ^∗^ *p* <0.05, ^∗∗^ *p* <0.01, ^∗∗∗^ *p* <0.001, ^∗∗∗∗^ *p* <0.0001 by Student’*s t*‐test.

It is widely acknowledged that liver pathologies can encompass various mechanisms of cellular demise. To identify the principal mechanisms of cell death in AS‐AIH, we developed murine models of AS‐AIH using concanavalin A (ConA) and administered inhibitors specific to apoptosis, necroptosis, pyroptosis, and ferroptosis—canonical forms of regulated cell death. Although various methodologies have been established to induce AIH in mice, the majority of these approaches are designed to mimic the chronic progression of the human disease, including the use of liver antigen preparations, *TGF‐β1* gene knockout, or thymectomy.^[^ ^17^, ^18^, ^19^ ^]^ However, ConA has the capacity to rapidly activate immune cells, leading to the generation of a plethora of inflammatory mediators that induce acute hepatic damage, and it represents the most frequently employed method for replicating the acute phase of human AIH.^[^ ^11^, ^20^ ^]^ The dosage of ConA used in the experiment was significantly higher than that in models of chronic and acute common hepatitis, ensuring that a large number of model mice succumbed within hours.^[^ ^21^, ^22^, ^23^ ^]^ Our findings revealed that Z‐VAD, an irreversible pan‐caspase inhibitor, exacerbated liver injury in AS‐AIH, a phenomenon consistent with prior research and potentially due to the inhibition of caspase redirecting TNF‐α signaling from apoptosis to necroptosis.^[^ ^9^, ^24^, ^25^ ^]^ Moreover, while the ferroptosis inhibitor UAMC‐3203 and the pyroptosis inhibitor VX‐765 have shown efficacy in acetaminophen‐induced and d‐galactosamine/lipopolysaccharide‐induced ALF in mice, respectively, they did not exhibit significant therapeutic benefits in AS‐AIH mice (Figure S1A,B, Supporting Information).^[^ ^26^, ^27^, ^28^, ^29^ ^]^ However, our study demonstrated that Nec‐1s, a necroptosis inhibitor, significantly ameliorated AS‐AIH, as indicated by a reduction in liver necrosis and decreased serum levels of alanine transaminase (ALT) and aspartate transaminase (AST) post‐treatment (**Figure** **1**A–C). This suggests that necroptosis plays a role in the pathogenesis of AS‐AIH.

To investigate the major cell populations that react to necroptosis during this process, we analyzed a set of single‐cell data from similar animal models in Gene Expression Omnibus (GSE201006). The findings indicated a rise in immune cell infiltration within the liver following ConA treatment (Figure S2A–C, Supporting Information). Further analysis revealed that among all cell populations, macrophages exhibited the most substantial increase in *Mlkl* expression, which serves as a marker for necroptosis (Figure 1D). Subsequently, we concentrated on macrophages and identified four distinct clusters (Figure 1E). Interestingly, unlike the other three clusters, the macrophages in cluster 2 exhibited minimal expression of *Mlkl* (Figure 1F). Therefore, we designated the second cluster as the MLKL‐negative cluster, while the remaining three clusters were termed the MLKL‐positive cluster. We discovered that *Tnfrsf1a* and *Ifngr1*, two key cytokine receptors that induce necroptosis, were upregulated in the MLKL‐positive clusters (Figure 1G). Additionally, the MLKL‐positive clusters exhibited a heightened state of immune activation, characterized by increased synthesis of cytokines and chemokines, as well as enrichment in numerous immune activation pathways (Figure 1H; Figure S2D, Supporting Information). To validate the results from single‐cell data, we utilized immunofluorescence (IF) staining to assess the expression of phosphorylated MLKL (P‐MLKL), a necroptosis marker, in the liver, with macrophages labeled using F4/80. The results confirmed the occurrence of necroptosis in macrophages during AS‐AIH, and its inhibition was evident with Nec‐1s treatment (Figure S2E, Supporting Information). Finally, flow cytometry (FC) analysis was applied to quantify the proportion of macrophages within non‐parenchymal cells in the liver, and enzyme‐linked immunosorbent assay (ELISA) was employed to determine the concentration of TNF‐α and interleukin 6 (IL‐6) in serum. We observed an increase in the proportion of macrophages following Nec‐1s treatment, coinciding with a decrease in the concentration of inflammatory factors (Figure 1I; Figure S2F, Supporting Information). These observations suggest that the necroptosis of inflammatory macrophages leads to the chaotic release of numerous inflammatory factors and chemokines, further exacerbating inflammatory damage. This may be the key factor in the progression to AS‐AIH. Targeting this mechanism to inhibit intrahepatic immune overactivation helps alleviate immune‐related liver injury.

### 2.2 MSCs Suppress Macrophage Necroptosis by Promoting the Transcription of *Sk1* in the Treatment of AS‐AIH

**Figure 2:**
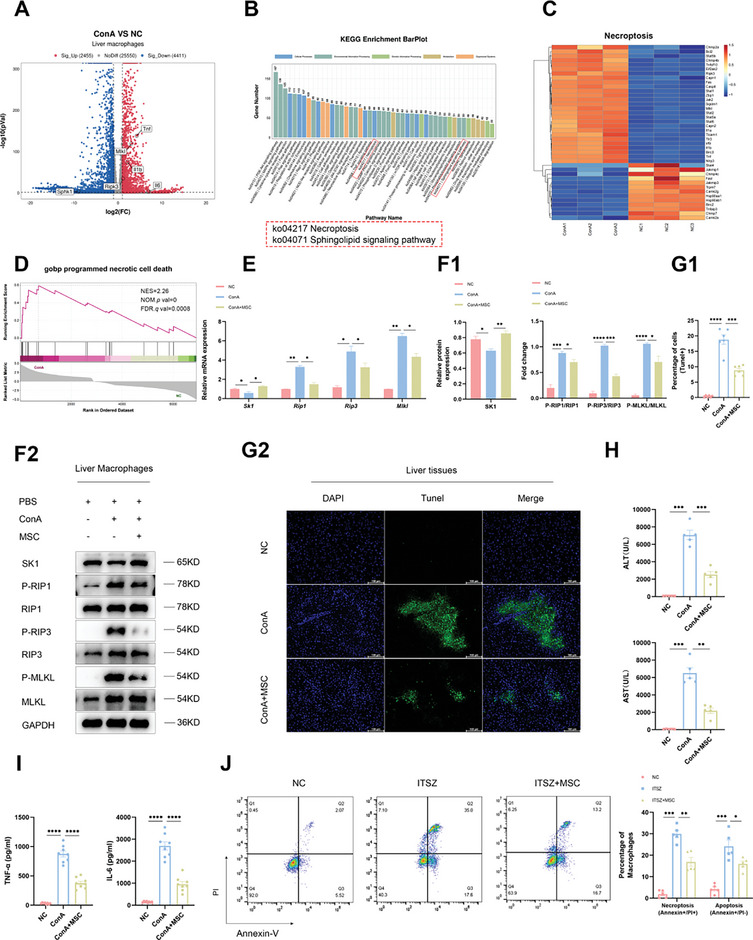
MSCs mitigate necroptosis of macrophages by promoting the transcription of *Sk1* in the treatment of AS‐AIH. A) Volcano map of DEGs in intrahepatic macrophages between NC and ConA groups (*n* = 3). B) KEGG enrichment analysis showed significant regulation of the necroptosis and sphingolipid signaling pathways in intrahepatic macrophages during AS‐AIH. C) Heatmap of DEGs in the necroptosis signaling pathway. D) GSEA plot of programmed necrotic cell death. E,F) ConA was employed to establish a mouse model of AS‐AIH, while MSCs were utilized for treatment. The mRNA and protein expressions of SK1 and necroptosis signaling pathways in intrahepatic macrophages were measured (*n* = 3). G) Tunel assay of apoptotic hepatocytes proportion in the liver (scale bars, 100 µm) *(n* = 6). H) Serum levels of ALT and AST were detected by ELISA (n = 5). I) Serum levels of TNF‐α and IL‐6 were detected by ELISA (*n* = 8). J) FC analysis of necrotic (Annexin‐V+/PI+) and apoptotic (Annexin‐V+/PI‐) proportion in mouse primary macrophages (*n* = 5). Data are presented as means ± SEM, ^∗^ *p* <0.05, ^∗∗^ *p* <0.01, ^∗∗∗^ *p* <0.001, ^∗∗∗∗^ *p* <0.0001 by Student’*s t*‐test.

To delve deeper into the gene expression dynamics of macrophages during AS‐AIH, we induced a model using ConA and dissociated the liver tissue after 12 h. Finally, macrophages (F4/80+) sorted via FC were subjected to RNA sequencing (RNA‐seq) analysis. The findings indicated elevated mRNA expressions of inflammatory factors, including *Tnf‐α, Il‐6, and Il‐1β*, along with necroptosis‐related genes in intrahepatic macrophages following ConA treatment (**Figure** **2**A). Further analysis demonstrated significant regulation of both necroptosis and sphingolipid signaling pathways during this period (Figure 2B–D). Interestingly, sphingosine‐1‐phosphate (S1P) has previously demonstrated a protective effect against ConA‐induced liver injury in mice.^[^ ^30^ ^]^ Therefore, we conducted a screening of differentially expressed genes (DEGs) within this pathway, revealing a significant inhibition in the transcription of sphingosine kinase 1 (SK1)  . As a key enzyme in the catalytic synthesis of S1P,^[^ ^31^ ^]^ we designed experiments to investigate its potential association with necroptosis and whether it could serve as a therapeutic target for MSCs. The MSCs used in the experiment were derived from mouse bone marrow (Figure S2G, Supporting Information). Quantitative real‐time polymerase chain reaction (qRT‐PCR) and Western blotting (WB) results revealed that MSCs treatment increased *Sk1* transcription in intrahepatic macrophages during AS‐AIH, accompanied by reduced synthesis and phosphorylation levels of RIPK1, RIPK3, and MLKL (Figure 2E,F). Terminal deoxynucleotidyl transferase dUTP nick‐end labeling (TUNEL) assay and ELISA analysis demonstrated a decrease in the proportion of apoptotic hepatocytes and serum liver enzyme as well as inflammatory cytokine levels following MSCs treatment (Figure 2G–I). In vitro, primary macrophages were treated with IFN‐γ, TNF‐α, SM164, and Z‐VAD (ITSZ) to induce necroptosis while co‐cultured with MSCs as the treatment group. FC analysis indicated that MSCs treatment effectively reduced the levels of cell necroptosis and apoptosis induced by ITSZ (Figure 2J). These findings indicate that the restricted transcription of *Sk1* impairs macrophage's ability to resist necroptosis. MSCs exhibit the capability to reverse this phenomenon, offering a potential therapeutic approach for AS‐AIH.

### 2.3 SP1 Participates in the Process of Macrophage Necroptosis by Modulating the Transcription of *Sk1*

**Figure 3:**
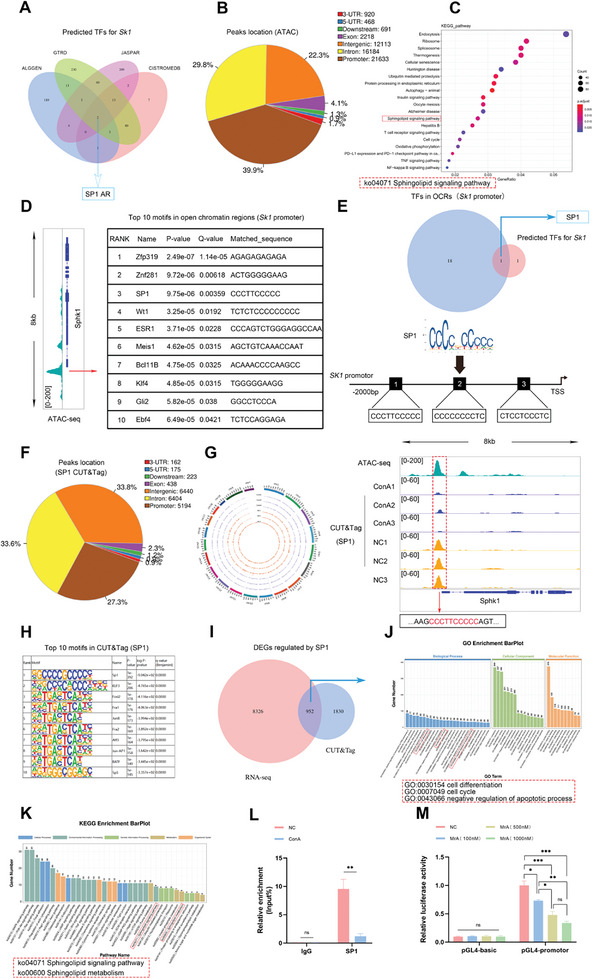
SP1 participates in the process of macrophage necroptosis by modulating the transcription of *Sk1*. A) Predicted TFs potentially binding to mouse *Sk1* promoter region (2kb upstream of transcription start site) as identified through publicly available databases. B) Locations of OCRs in mouse intrahepatic macrophages identified through ATAC‐seq analysis. C) KEGG enrichment analysis based on the gene ratio identified in ATAC‐seq data. D) The top 10 motifs found within OCRs of mouse intrahepatic macrophage *Sk1* promoter. E) SP1 emerged as the primary candidate for regulating *Sk1* transcription in mouse intrahepatic macrophages through the integrated analysis of ATAC‐seq data and public databases. F) The peak distribution detected in CUT&Tag analysis of mouse intrahepatic macrophages. G) The regulatory role of SP1 in *Sk1* transcription within mouse intrahepatic macrophages was demonstrated through the integrated analysis of ATAC‐seq and CUT&Tag data (*n* = 3). H) The top ten motifs identified in CUT&Tag analysis. I) DEGs regulated by SP1 were confirmed by the integrated analysis of CUT&Tag and RNA‐seq data (*n* = 3). J,K) GO and KEGG enrichment analysis of DEGs regulated by SP1. L) The detection of SP1‐mediated regulation of *Sk1* transcription in mouse intrahepatic macrophages was achieved through ChIP‐qRT‐PCR (*n* = 3). M) The capability of SP1 to regulate *Sk1* transcription in vitro was validated by treating mouse primary macrophages with varying concentrations of MrA and assessing luciferase activity *(n* = 3). Data are presented as means ± SEM, ^∗^ *p* <0.05, ^∗∗^ *p* <0.01, ^∗∗∗^ *p* <0.001 by Student’*s t*‐test and one‐way ANOVA with Tukey's post hoc test.

To investigate why *Sk1* transcription is suppressed during necroptosis in intrahepatic macrophages of AS‐AIH mice, our focus turned to transcription factors (TFs), recognized as key regulators of gene expression. Through an analysis of various public databases aimed at predicting TFs for mouse *Sk1*, we identified specificity protein 1 (SP1) and androgen receptor as consistently appearing TFs across all databases examined (**Figure** **3**A). To refine our selection of TF for subsequent studies, we performed an assay for transposase accessible chromatin sequencing (ATAC‐seq) analysis on sorted mouse intrahepatic macrophages (Figure 3B). The findings indicated that sphingolipid signaling is essential for the physiological function of intrahepatic macrophages (Figure 3C). Further analysis revealed the existence of open chromatin regions (OCRs) within the *Sk1* promoter. By subsequently identifying motifs within OCRs and conducting joint analysis with TFs predicted by the database, SP1 emerged as the prime candidate for further investigation (Figures 3D,E).

To explore the alterations in the capacity of SP1 in regulating transcription in mouse intrahepatic macrophages during AS‐AIH, we conducted cleavage under targets and tagmentation (CUT&Tag) assays on intrahepatic macrophages obtained from both normal and AS‐AIH mice (Figure 3F). The findings indicated that the promoter region of *Sk1* exhibits peaks at identical positions to OCRs identified in ATAC‐seq. Additionally, a trend toward a decrease in peak intensity was observed in the ConA‐induced AS‐AIH group (Figure 3G,H). Subsequently, we integrated the CUT&Tag and RNA‐seq data from the two groups of cells to identify DEGs that could be influenced by the altered regulatory capacity of SP1 (Figure 3I). Enrichment analysis based on these genes indicated that SP1 exerts significant influence over the differentiation, cell cycle regulation, and anti‐apoptotic mechanisms within intrahepatic macrophages (Figure 3J). These findings align with the previous conclusion that SP1 is an indispensable TF for cells to perform their physiological functions.^[^ ^32^ ^]^ Furthermore, SP1 was noted to be significant in both the sphingolipid signaling pathway and sphingolipid metabolism (Figure 3K). To further corroborate the capacity of SP1 in regulating *Sk1* transcription, we conducted qRT‐PCR analysis on DNA extracted from chromatin immunoprecipitation (ChIP) assays involving intrahepatic macrophages in both models. Primers were tailored to the sequence encompassing the SP1 binding site within the peak of the *Sk1* promoter region in both ATAC‐seq and CUT&Tag. The results demonstrated that ConA treatment reduced the binding affinity of SP1 to *Sk1* promoter in intrahepatic macrophages (Figure 3L). Finally, mouse primary macrophages were transfected with a luciferase reporter plasmid harboring the *Sk1* promoter sequence and subsequently treated with MrA at various concentrations. Luciferase activity was then assessed to validate the role of SP1 in regulating *Sk1* transcription in vitro (Figure 3M). The findings from multi‐omics analysis, coupled with experimental validation, demonstrated that SP1 within intrahepatic macrophages possesses the capability to positively regulate *Sk1* transcription. However, this regulatory capacity is hindered during necroptosis of intrahepatic macrophages in AS‐AIH mice.

### 2.4 MSCs Exert Therapeutic Effects by Delivering SP1 to Facilitate the Transcription of *Sk1* in Macrophages

Based on the regulatory role of SP1 in *Sk1* transcription within macrophages, we postulated that MSCs could potentially suppress macrophage necroptosis via modulation of the SP1/SK1 axis. Therefore, we tested this hypothesis in the MSC‐treated group by using MrA, an SP1 inhibitor, and PF543, an SK1 inhibitor. In vitro experiments unveiled a decline in the protein levels of SP1 and SK1 during macrophage necroptosis, a trend that was reversed upon MSCs treatment. However, inhibitors significantly diminished the therapeutic effect of MSCs. A similar trend was observed at the transcriptional level (Figure S3A,C, Supporting Information). Notably, only the transcriptional activity of *Sk1* was heightened in MSCs treatment group (Figure S3C, Supporting Information). These observations suggested that MSCs may increase the protein level of SP1 in macrophages through other mechanisms. MSCs often exert their therapeutic effects by secreting biologically active substances.^[^ ^33^ ^]^ Therefore, we conducted a WB analysis of molecules present in MSCs supernatant, uncovering the presence of SP1 (Figure S4A, Supporting Information). This suggested that MSCs may enhance the transcription of *Sk1* in macrophages by delivering SP1, thereby improving their resistance to necroptosis.

**Figure 4:**
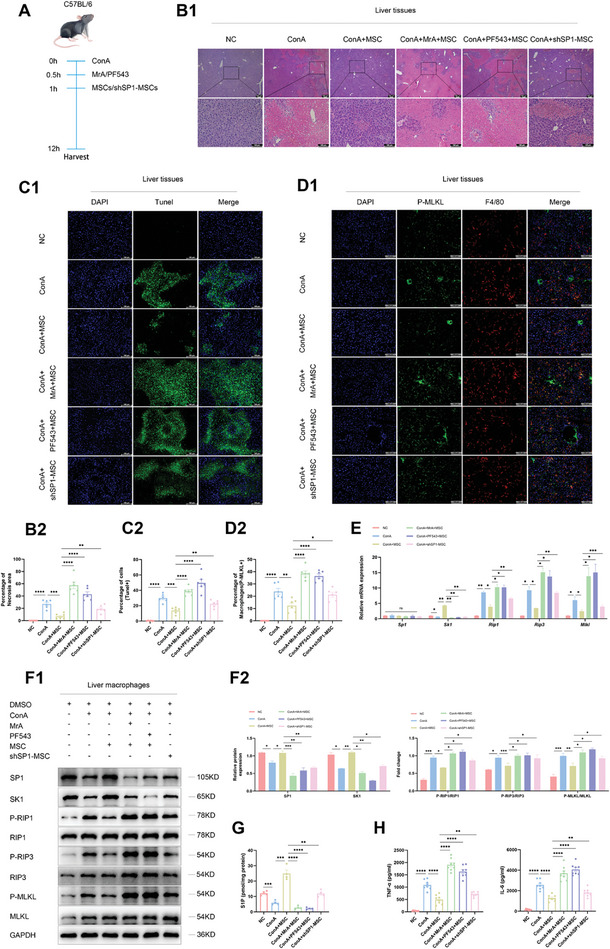
MSCs suppress macrophage necroptosis by upregulating the SP1/SK1/S1P axis as a therapeutic strategy for AS‐AIH. A) Schematic of the setup for inducing AS‐AIH and treatment model in C57/BL6 mice. B) Representative H&E stained images showed the histological morphology of liver tissues (scale bars, 100 µm) (*n* = 6). C) Tunel assay of apoptotic hepatocytes proportion in liver (scale bars, 100 µm) (*n* = 6). D) Representative IF images showed the expression of P‐MLKL in intrahepatic macrophages (scale bars, 100 µm) (*n* = 6). E) The mRNA levels of *Sp1, Sk1, Rip1, Rip3*, and *Mlkl* in intrahepatic macrophages (*n* = 3). F) WB analysis of SP1/SK1 axis and necroptosis signaling pathway in intrahepatic macrophages (*n* = 3). G) S1P concentrations in intrahepatic macrophages were detected by ELISA (*n* = 4). H) Serum levels of TNF‐α and IL‐6 were detected by ELISA (*n* = 8). Data are presented as means ± SEM, ^∗^ *p* <0.05, ^∗∗^ *p* <0.01, ^∗∗∗^ *p* <0.001, ^∗∗∗∗^ *p* <0.0001 by Student’*s t*‐test.

Following this, we utilized lentivirus carrying short hairpin RNA targeting SP1 for MSCs to validate the hypothesis (Figure S4B, Supporting Information). We found that MSCs lacking endogenous SP1 showed a significantly weakened therapeutic effect, due to their limited ability to promote macrophage *Sk1* transcription (Figure S3B,C, Supporting Information). Luciferase activity assays conducted on macrophages within each group provided further confirmation that the delivery of SP1 by MSCs reversed the transcriptional restriction of *Sk1* during necroptosis (Figure S3D, Supporting Information). IF staining also revealed an inverse relationship between the co‐expression of SP1 and P‐MLKL in macrophages (Figure S3E, Supporting Information). FC analysis indicated that MSCs treatment effectively lowered the proportion of macrophage necrosis and apoptosis induced by ITSZ. However, the treatment efficacy was hindered in both the inhibitor and lentivirus groups (Figure S3F, Supporting Information). To examine whether MSCs could exert their therapeutic effects in vivo through the aforementioned mechanisms, we applied the same treatment to ConA‐induced AS‐AIH mice (**Figure** **4**A). H&E staining and TUNEL assay of liver tissues, as well as survival curve analysis collectively demonstrated that the therapeutic efficacy of MSCs was diminished following the combination of inhibitor treatment or SP1 knockdown by lentivirus (Figure 4B,C; Figure S4C, Supporting Information).

In terms of mechanism, IF staining was employed to value the level of P‐MLKL in macrophages within liver tissues (Figure 4D). Additionally, qRT‐PCR and WB analyses were conducted to assess the level of the SP1, SK1, and molecules associated with the necroptosis signaling pathway in sorted liver macrophages (Figure 4E,F). Consistent with the trend in vitro, MSCs treatment activated the SP1/SK1 axis within intrahepatic macrophages by delivering SP1, thereby suppressing necroptosis. ELISA analysis revealed that the concentration of S1P, the end product resulting from catalysis of SK1, in intrahepatic or primary macrophage lysates decreased in the injury group and increased following MSCs treatment. However, this increase was reversed in both the inhibitor group and the lentivirus group (Figure 4G; Figure S3G, Supporting Information). The parallel trajectories observed for both SK1 and S1P reaffirmed the significant role of SK1 in macrophages during the disease process. Finally, the concentrations of inflammatory factors and ALT/AST, detected by ELISA suggested that macrophage necroptosis results in elevated levels of inflammatory factors, subsequently contributing to aggravated liver injury (Figure 4H; Figures S3H and S4D, Supporting Information). To further ascertain the therapeutic impact of SP1 as a key paracrine factor, we conducted an experiment overexpressing SP1 in MSCs. The findings indicate that this overexpression enhances MSCs' ability to modulate the SK1/S1P axis in macrophages, resulting in reduced serum levels of pro‐inflammatory factors and mitigated liver injury in the murine model (Figure S4E–G, Supporting Information). Taken together, these studies demonstrate that MSCs regulate macrophage *Sk1* transcription via SP1 delivery, thereby inhibiting necroptosis and alleviating liver injury induced by excessive immunization.

### 2.5 MSCs Facilitate the Transition of Macrophages from M1 to M2 Phenotype During AS‐AIH

**Figure 5:**
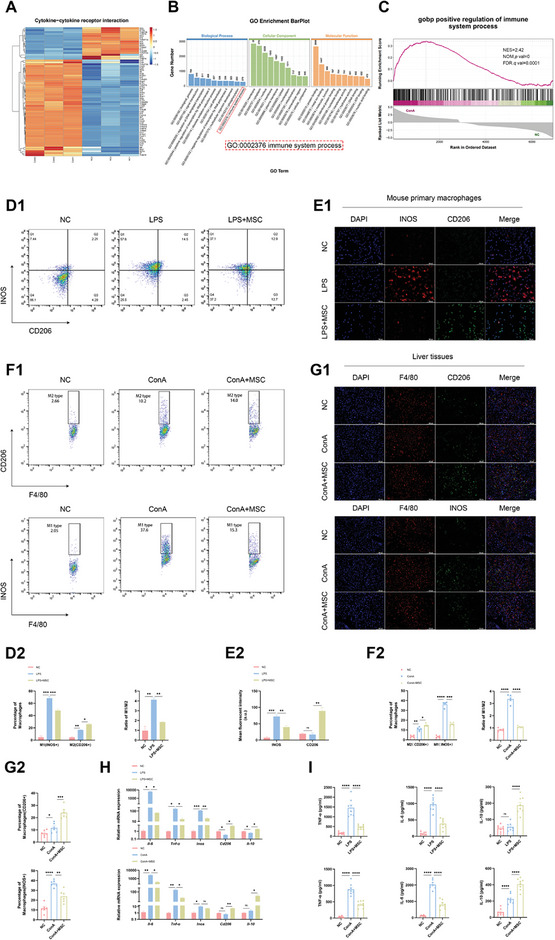
MSCs facilitate the transition of macrophages from M1 to M2 phenotype during AS‐AIH. A) Heatmap of DEGs in cytokine‐cytokine receptor interaction. B) GO enrichment analysis indicated that macrophages played a role in regulating the intrahepatic immune response during AS‐AIH. C) GSEA of gobp revealed that macrophage actively regulated immune processes. D) FC analysis of INOS and CD206 expression in mouse primary macrophages (*n* = 3). E) Representative IF images showed the expression of INOS and CD206 in mouse primary macrophages (scale bars, 100 µm) (*n* = 3). F) FC analysis of INOS and CD206 expression in intrahepatic macrophages (*n* = 5). G) Representative IF images showed the expression of INOS and CD206 in intrahepatic macrophages (scale bars, 100 µm) (*n* = 6). H) The mRNA levels of *Il‐6, Tnf‐α, Inos, Cd206*, and *Il‐10* in mouse primary and intrahepatic macrophages (*n* = 3). I) TNF‐α, IL‐6, and IL‐10 concentrations in macrophage supernatants and serum were detected by ELISA (*n* = 8). Data are presented as means ± SEM, ^∗^ *p* <0.05, ^∗∗^ *p* <0.01, ^∗∗∗^ *p* <0.001, ^∗∗∗∗^ *p* <0.0001 by Student’*s t*‐test.

As the initial responders to tissue injury, macrophages hold a crucial position in AS‐AIH. RNA‐seq analyses revealed that macrophages were in an activated inflammatory state during this period, characterized by heightened transcription of cytokine and receptors (**Figure** **5**A–C). The potent immunomodulatory capabilities of MSCs are widely recognized, primarily characterized by their significant anti‐inflammatory effects.^[^ ^34^ ^]^ Subsequently, we proceeded to confirm the impact of MSCs on macrophage polarization. In vitro, FC and IF staining demonstrated that combined MSCs treatment resulted in reduced inducible nitric oxide synthase (INOS) expression and increased mannose receptor (CD206) expression compared to M1 polarization induced by lipopolysaccharide (Figure 5D,E). In vivo, similar trends were observed when treating AS‐AIH mice with MSCs and repeating the aforementioned experiments (Figure 5F,G). Similar alterations in mRNA levels of *Inos* and *Cd206* were likewise observed in macrophages (Figure 5H). Of utmost significance, we discovered that the mRNA levels of *Tnf‐α* and *Il‐6* in pro‐inflammatory states, along with their concentrations in supernatant and serum, decreased following MSCs treatment. Additionally, anti‐inflammatory cytokine IL‐10 exhibited an opposite trend (Figure 5H,I). Hence, we propose that MSCs suppress necroptosis of macrophages while simultaneously reversing their proinflammatory state.

### 2.6 S1P Promotes Hepatocyte Proliferation by Modulating YAP Signaling via S1PR2

**Figure 6:**
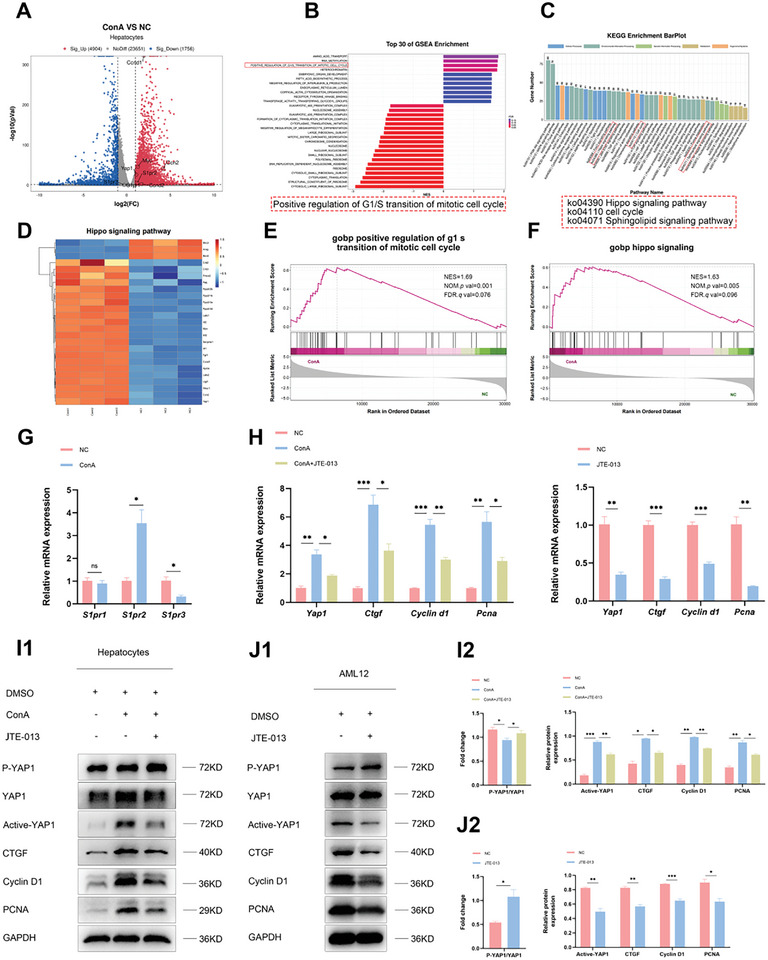
S1P promotes hepatocyte proliferation by modulating the Hippo/YAP signaling pathway via S1PR2. A) Volcano map of DEGs in hepatocytes between NC and ConA groups (*n* = 3). B) The top 30 results from GSEA suggested the heightened proliferative state of hepatocytes during AS‐AIH. C) KEGG enrichment analysis showed significant regulation of the hippo and sphingolipid signaling pathways in hepatocytes during AS‐AIH. D) Heatmap of DEGs in the hippo signaling pathway. E,F) GSEA of gobp indicated that hepatocytes entered a proliferative state during AS‐AIH, with significant regulation of the hippo signaling. G) The mRNA levels of *S1pr1, S1pr2, and S1pr3* in hepatocytes in vivo (*n* = 3). H) The mRNA levels of *Yap1, Ctgf, Cyclin d1, and Pcna* in hepatocytes in vivo and AML12 cells (*n* = 3). I,J) WB analysis of YAP signaling pathway in hepatocytes in vivo and AML12 cells (*n* = 3). Data are presented as means ± SEM, ^∗^ *p* <0.05, ^∗∗^ *p* <0.01, ^∗∗∗^ *p* <0.001 by Student’*s t*‐test.

The majority of hepatocytes in the mature normal liver are in a state of proliferative quiescence. However, various factors can lead to liver damage, triggering the initiation of liver regeneration mechanisms.^[^ ^35^ ^]^ To investigate liver regeneration during AS‐AIH, we collected mouse liver 36 h after modeling and isolated hepatocytes for RNA‐seq analysis. We observed that DEGs associated with the cell cycle were notably enriched in hepatocytes following injury, indicating a re‐entry into a proliferative state. Further analyses revealed significant enrichment of both the Hippo/YAP and sphingolipid signaling pathways in this process (**Figure** **6**A–F). Previous studies have shown that S1P regulates YAP signaling, leading to disordered hepatocyte proliferation and contributing to the development of hepatocellular carcinoma.^[^ ^36^ ^]^ As a vital bioactive sphingolipid, S1P demonstrates a range of positive effects, including cell survival, proliferation, and migration via specific S1P receptors (S1PR1‐5), in contrast to other molecules in sphingolipid signaling.^[^ ^37^, ^38^ ^]^ With S1PR4/5 primarily expressed in the nervous system and immune system, our investigation centered on analyzing the alterations in the transcriptional levels of *S1pr1‐3* within hepatocytes.^[^ ^38^ ^]^ Finally, only *S1pr2* transcription was found to be upregulated, which was also confirmed by subsequent qRT‐PCR experiments (Figure 6A,G).

To further confirm whether S1P regulates YAP signaling in hepatocytes via S1PR2 and participates in liver regeneration during AS‐AIH, we utilized the specific inhibitor of S1PR2, JTE‐013, for validation. Through qRT‐PCR and WB experiments, it was discovered that during liver injury, there was an increase in synthesis and a decrease in phosphorylation of YAP in hepatocytes. This resulted in the activation of regenerative pathways and the promotion of liver proliferation. The combination of JTE‐013 attenuated this effect (Figure 6H,I). JTE‐013 also showed the same effect on AML12 cells in vitro (Figure 6H,J). Hence, targeting the S1P/S1PR2/YAP axis to promote hepatocyte proliferation may emerge as a crucial approach for the treatment of AS‐AIH.

### 2.7 MSCs Regulate YAP Signaling in Hepatocytes to Promote Liver Regeneration by Enhancing S1P Synthesis in both Hepatocytes and Macrophages

**Figure 7:**
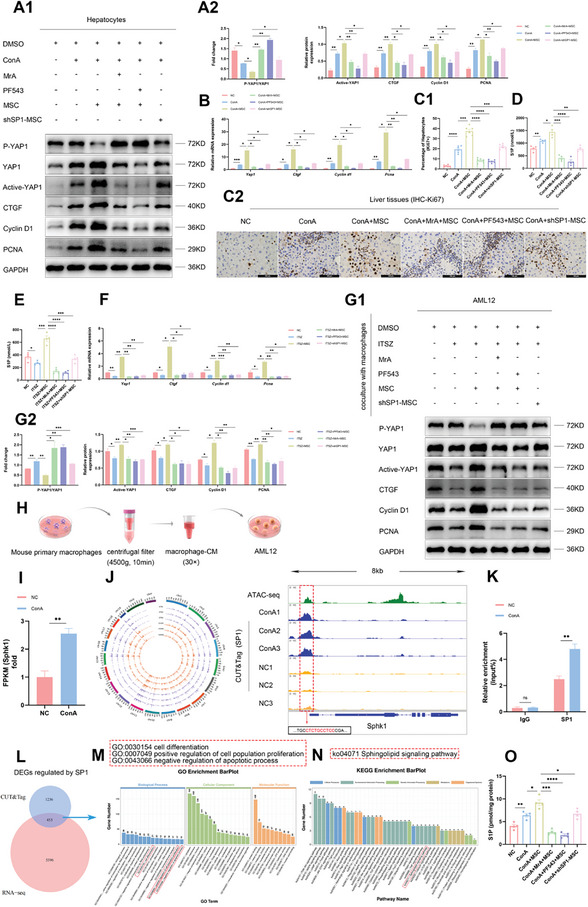
MSCs regulate YAP signaling in hepatocytes to promote liver regeneration by enhancing S1P synthesis in both hepatocytes and macrophages. A) WB analysis of YAP signaling pathway in hepatocytes in vivo (*n* = 3). B) The mRNA levels of *Yap1, Ctgf, Cyclin d1, and Pcna* in hepatocytes in vivo (*n* = 3). C) Representative IHC images showed the Ki67‐positive hepatocytes proportion in liver tissues (scale bars, 100 µm) (*n*=6). D, E) S1P concentrations in serum and macrophage supernatants were detected by ELISA (*n*=4). F) The mRNA levels of *Yap1, Ctgf, Cyclin d1, and Pcna* in AML12 cells (*n* = 3). G) WB analysis of YAP signaling pathway in AML12 cells (*n* = 3). H) Schematic illustration of an in vitro experiment wherein secretion from macrophages was applied to hepatocyte culture. I) The relative expression of *Sk1* in hepatocytes from two groups (*n* = 3). J) The regulatory role of SP1 in *Sk1* transcription within hepatocytes was demonstrated through the integrated analysis of ATAC‐seq and CUT&Tag data (*n* = 3). K) The detection of SP1‐mediated regulation of *Sk1* transcription in hepatocytes was achieved through ChIP‐qRT‐PCR (*n* = 3). L) DEGs regulated by SP1 were confirmed by the integrated analysis of CUT&Tag and RNA‐seq data (*n* = 3). M, N) GO and KEGG enrichment analysis of DEGs regulated by SP1. O) S1P concentrations in hepatocytes were detected by ELISA (*n* = 4). Data are presented as means ± SEM, ^∗^ *p* <0.05, ^∗∗^ *p* <0.01, ^∗∗∗^ *p* <0.001, ^∗∗∗∗^ *p* <0.0001 by Student’*s t‐*test.

Considering the significant therapeutic role of S1P in both macrophages and hepatocytes during AS‐AIH, we hypothesized that MSCs‐mediated delivery of SP1 to regulate the SK1/S1P axis would also hold a substantial effect on liver regeneration. Hence, the in vivo validated model of macrophage necroptosis mentioned above was established, and this time the liver was collected after 36 h to isolate hepatocytes for experiments. Results demonstrated that MSCs treatment augmented the proliferative effect of YAP signaling on hepatocytes, as evidenced by increased synthesis and reduced phosphorylation of YAP as well as the activation of downstream signals. Inhibitor or lentivirus resulted in the inhibition of this therapeutic effect (**Figure** **7**A,B). The immunohistochemical detection of Ki67 expression in hepatocytes within liver tissue exhibited a similar trend (Figure 7C). ELISA assay indicated that MSCs further elevated the S1P concentration in serum, but this effect was significantly attenuated by inhibitors and lentivirus (Figure 7D). These validate the dual therapeutic impact of MSCs targeting the SP1/SK1/S1P axis in AS‐AIH.

Previous studies have shown that S1P mainly exerts its biological functions through autocrine, paracrine, and endocrine pathways.^[^ ^39^ ^]^ Given that MSCs can inhibit necroptosis by promoting S1P synthesis in macrophages during AS‐AIH. We speculated that this might synergistically promote hepatocyte proliferation. Following this, we removed the media from the macrophage necroptosis and MSCs treatment groups, replaced it with fresh media, and continued the culture for 24 h. ELISA was used to assess the S1P concentration in the supernatant. Results confirmed that macrophages could release S1P. MSCs enhanced this effect, but it was significantly inhibited by inhibitors and lentivirus (Figure 7E).

Subsequently, a concentrated macrophage‐conditioned medium (macrophage‐CM) was introduced into the AML12 medium. QRT‐PCR and WB results indicated that YAP signaling was modulated in line with the observed trend in S1P concentration within macrophage‐CM (Figure 7F–H). Similarly, IF staining indicated that macrophage‐CM derived from MSCs group significantly increased the content and facilitated nuclear localization of YAP within AML12 cells compared to other groups (Figure S5A, Supporting Information). This regulatory effect was also validated through cell proliferation assays (Figure S5B–E, Supporting Information). These confirm our aforementioned conjecture. Interestingly, the serum S1P concentration in the ConA group was higher than that in the NC group, despite necroptosis of intrahepatic macrophages after ConA treatment leading to limited S1P synthesis (Figure 7D,E). Therefore, we suggest that other sources of S1P are present to support hepatocyte proliferation during AS‐AIH. As target cells, we initially investigated the ability of hepatocytes to synthesize S1P. RNA‐seq analysis indicated that the transcription of *Sk1* was enhanced following ConA treatment (Figure 7I).

Considering the crucial role of the SP1/SK1/S1P axis in liver regeneration, we conducted ATAC‐seq and CUT&Tag on isolated hepatocytes. Enrichment analysis of genes with OCRs in hepatocytes indicated that the sphingolipid signaling pathway was vital for hepatocyte function (Figure S6A,B, Supporting Information). OCR was identified in the promoter region of *Sk1* in hepatocytes, located similarly to that in macrophages (Figure 7J). To explore whether SP1 is involved in regulating hepatocyte *Sk1* transcription during AS‐AIH, we conducted CUT&Tag assays on mouse hepatocytes from both the NC and ConA groups (Figure S6C,D, Supporting Information). The results indicated that ConA treatment led to an increased transcriptional effect of SP1 on *Sk1* in hepatocytes compared to the NC group (Figure 7J).

Following this, we conducted ChIP assays using SP1 antibody on both sets of hepatocytes. ChIP‐qRT‐PCR results demonstrated a similar trend to the CUT&Tag findings (Figure 7K). To investigate the genes regulated by SP1 during this period, we integrated the CUT&Tag and RNA‐seq data from both groups of hepatocytes (Figure 7L). Enrichment analysis indicated that SP1 was crucial for hepatocyte differentiation, proliferation, and anti‐apoptotic processes (Figure 7M). Notably, the sphingolipid signaling pathway was regulated by SP1 (Figure 7N). The concentration of S1P in hepatocyte lysates in each group suggested that ConA treatment promoted S1P synthesis in hepatocytes. MSCs targeting the SP1/SK1/S1P axis further amplified this effect (Figure 7O).

To verify the role of the SP1/SK1 axis in regulating YAP signaling in hepatocyte proliferation, lentivirus was used to inhibit SP1 expression in AML12 cells. WB, qRT‐PCR, and ELISA results demonstrated that inhibiting SP1 expression led to the suppression of the SK1/S1P/YAP axis in hepatocytes (Figure S6E,G,I, Supporting Information). Luciferase activity assay further confirmed that inhibiting SP1 expression led to restricted SK1 transcription in AML12 cells (Figure S6J, Supporting Information). To verify whether MSCs can target this pathway to promote hepatocyte proliferation by delivering SP1. We introduced MSC‐CM into the AML12 medium and compared it with MSCs that used lentivirus to inhibit SP1 expression. WB, qRT‐PCR, ELISA, and luciferase activity assay results indicated that MSCs targeting the SP1/SK1/S1P axis promoted YAP signaling‐mediated hepatocyte proliferation. Inhibiting SP1 expression in MSCs restricted this function (Figure S6F, H‐J, Supporting Information). A similar trend was also observed in the cell proliferation assays (Figure S6K–N, Supporting Information).

**Figure 8:**
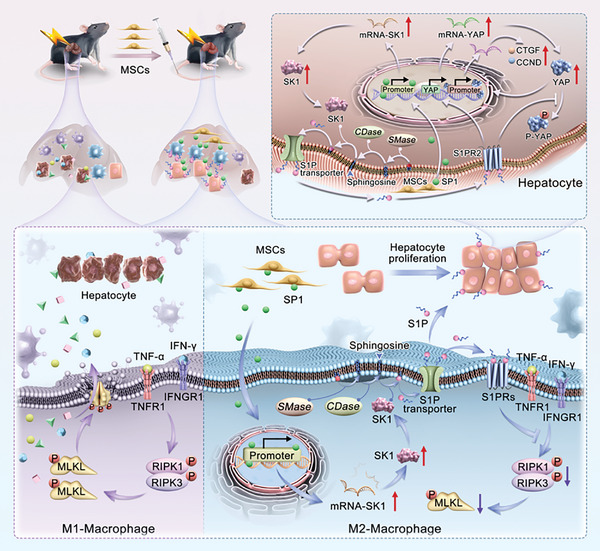
Schematic representation of how MSCs modulate macrophage fate to alleviate liver damage and promote liver regeneration in the treatment of AS‐AIH. MSCs up‐regulate the SP1/SK1/S1P axis through SP1 delivery to inhibit macrophage necroptosis. Simultaneously, they promote M2 polarization of macrophages, further reducing inflammatory injury. Furthermore, The SP1/SK1 axis in hepatocytes controls S1P synthesis, promoting liver regeneration by regulating YAP signaling through S1PR2. MSCs target the SP1/SK1 axis to enhance S1P synthesis in macrophages and hepatocytes, thereby promoting liver regeneration.

Furthermore, we generated mice with hepatocyte‐specific *S1pr2* deletion to elucidate the role of liver regeneration in AS‐AIH. By inducing AS‐AIH in both control and *S1pr2* conditional knockout (cKO) mice and administering MSCs, we observed that hepatocytes in cKO mice were virtually incapable of activating YAP signaling upon liver injury, irrespective of MSC treatment. Notably, the expression of YAP signaling‐associated proteins and mRNA, along with those linked to its downstream proliferative pathway, were substantially reduced in cKO hepatocytes relative to controls. A congruent pattern was noted in Ki67 expression within hepatocytes of liver tissue sections. These observations underscore the pivotal function of the S1PR2‐YAP axis in hepatic regeneration during AS‐AIH (Figure S7A–E, Supporting Information). Consequently, we hypothesize that MSCs augment S1P synthesis in macrophages through SP1 delivery, thereby inhibiting necroptosis and bolstering liver regeneration synergistically. Hepatic regeneration in AS‐AIH is predominantly governed by the SP1/SK1/S1P/YAP axis within hepatocytes. MSCs influence macrophages and directly target this axis to foster hepatocyte proliferation (**Figure** **8**).

## 3 Discussion

AIH is a type of liver injury caused by immune system abnormalities with an unknown etiology. It is typically characterized by the presence of autoantibodies, elevated liver enzymes, and IgG levels.^[^ ^40^ ^]^ In fact, the clinical presentation of AIH varies greatly, ranging from no symptoms to liver failure. AS‐AIH refers to acute severe hepatitis, either with a history of AIH or without any apparent cause. Due to the lack of AIH typical characteristics, it is often diagnosed by exclusion, and the actual incidence may be higher than reported. As a result, conventional AIH treatments, such as corticosteroids or immunosuppressants, can only produce limited curative effects.^[^ ^41^ ^]^ Limited treatment options and rapid disease progression result in a very poor prognosis for AS‐AIH. Studies have shown that the effectiveness rate of conventional treatment for AS‐AIH is less than 15%, making liver transplantation often the only viable option. However, the autoimmune characteristics make it more prone to relapse after liver transplantation compared to other liver diseases.^[^ ^41^, ^42^ ^]^ Therefore, it is essential to clarify the pathogenesis of AS‐AIH and develop targeted therapies.

The severity of the injurious disease depends on the extent of cell death. Necroptosis has been identified as the primary mode of cell death in AIH, which may be related to the presence of a large number of cytokines.^[^ ^9^, ^10^, ^11^, ^12^ ^]^ The contents released after cell death further activate the intrahepatic immune system, potentially leading to ongoing exacerbation of the injury. However, it is unclear whether this phenomenon is involved in the development of AS‐AIH. In this study, we constructed a ConA‐induced AS‐AIH model and analyzed corresponding public single‐cell data. Our findings revealed that necroptosis is involved in the progression of AS‐AIH, as Nec‐1s can significantly alleviate the severity of liver injury. Further studies revealed that the main cell population undergoing necroptosis are macrophages, particularly those in an inflammatory activation state. As the primary initiators and coordinators of immune responses, macrophages can engulf pathogens, secrete a variety of cytokines and chemokines, and are also capable of recruiting and regulating other immune cells. Macrophages possess remarkable plasticity, allowing them to adopt diverse states in response to various stimuli.^[^ ^43^ ^]^ Research indicates that after post‐inflammatory activation, macrophages progressively diminish their secretion of pro‐inflammatory cytokines, with the M1‐to‐M2 phenotype transition typically concluding within 2 to 3 days.^[^ ^44^ ^]^ This orchestrated change in functional state is crucial for macrophages to modulate the immune milieu and facilitate tissue repair.^[^ ^45^, ^46^, ^47^, ^48^ ^]^ In addition to polarization, they are influenced by pathogen or inflammatory factors and may face the threat of cell death during the disease progression, ultimately resulting in aggravated tissue damage.^[^ ^49^, ^50^, ^51^ ^]^ Studies have shown that macrophage dysfunction is a key factor in the development of autoimmune diseases.^[^ ^52^ ^]^ However, as of now, the existence of this phenomenon during AS‐AIH has yet to be conclusively demonstrated. Our findings revealed that inhibiting macrophage necroptosis reduced serum levels of inflammatory factors. Given that necroptosis primarily occurs in inflammatory macrophages, we believe that it triggers the release of a substantial amount of inflammatory factors and chemokines, which exacerbates liver damage and promotes the ongoing recruitment of immune cells, including macrophages, into the liver. This process ultimately initiates a positive feedback loop of tissue damage and immune activation.

Sphingolipid signaling is crucial for cell function and state. S1P, a pivotal product of sphingolipid metabolism, has been demonstrated to exert crucial regulatory functions in immunity, cell cycle regulation, anti‐apoptosis mechanisms, and the promotion of regeneration.^[^ ^53^, ^54^, ^55^, ^56^ ^]^ Indeed, S1P has been demonstrated to prevent the transition from a pro‐survival state to a pro‐apoptotic state by inducing RIPK1 ubiquitination. Moreover, it has also been shown to mitigate ischemia‐reperfusion‐induced necrosis in renal cells.^[^ ^56^, ^57^ ^]^ As the key enzyme involved in S1P synthesis, the transcription of *Sk1* was markedly inhibited in macrophages from the AS‐AIH group. Nevertheless, it remains uncertain whether SK1 is involved in regulating macrophage necroptosis during AS‐AIH and its potential as a therapeutic target through MSCs intervention. In this study, we identified the critical role of SK1 in regulating macrophage necroptosis. MSCs suppressed the inflammatory response and ameliorated liver injury by enhancing the expression of SK1 to counter macrophage necroptosis. Nevertheless, further exploration is required to elucidate the precise mechanisms through which MSCs regulate the transcription of *Sk1*.

TFs, being pivotal determinants of gene transcription, have consistently remained a focal point in research endeavors. Research has indicated that bioactive substances delivered by MSCs can serve as TFs in target cells, either through direct action or indirect modulation.^[^ ^57^, ^58^, ^59^ ^]^ SP1, one of the earliest TFs identified in mammals, governs the transcriptional activity of numerous genes and plays a crucial role in regulating diverse biological functions within cells.^[^ ^60^, ^61^ ^]^ Here, we present evidence demonstrating that SP1 promotes *Sk1* transcription in mouse macrophages. This regulatory capacity is compromised during macrophage necroptosis, ultimately leading to the inhibition of S1P synthesis. Subsequent studies demonstrated that MSCs targeted this pathway by delivering SP1 to inhibit macrophage necroptosis during this period. In addition to their pro‐inflammatory role in the early stages, macrophages also contribute to tissue repair during the intermediate and later stages of liver injury diseases. Prevailing concepts attribute this phenomenon to the functional plasticity of macrophages, that is, the transition from a tissue‐destructive state to subsequent catabolic or tissue‐reparative properties.^[^ ^62^, ^63^ ^]^ Here, we discovered that MSCs suppressed macrophage necroptosis while simultaneously promoting its M2 polarization. This discovery appears to address one of the challenges in regulating macrophages for the treatment of acute liver injury. According to the previous perspective, excessive macrophage infiltration in the early stage of the disease worsens liver inflammatory injury. If clearance measures are taken, macrophage‐mediated liver regeneration in the middle and late stages will be inhibited.^[^ ^63^ ^]^ By modulating the fate of macrophages, MSCs can expedite the transition of the damaged liver into the repair state.

Robust regenerative capacity of the liver is essential for preserving a stable ratio relative to body weight. Liver volume typically restores to its pre‐injury level within a matter of weeks.^[^ ^64^ ^]^ Research has demonstrated that immune cells, particularly macrophages, facilitate liver regeneration either through direct contact or by delivering cytokines.^[^ ^64^, ^65^ ^]^ Interestingly, S1P is also implicated, although the precise mechanism remains unknown.^[^ ^66^ ^]^ In this study, RNA‐seq analysis uncovered that hepatocytes within damaged livers initiated proliferation, accompanied by significant regulation of hippo and sphingolipid signaling pathways. Involvement of the hippo signaling pathway in various physiological and pathological processes of the liver is well established. This includes its role in initial liver development, repair of damage, and even malignant transformation.^[^ ^67^ ^]^ Studies have indicated that S1P modulates the hippo signaling pathway, potentially contributing to the uncontrolled proliferation of hepatocytes.^[^ ^36^ ^]^ Our research pinpointed S1PR2 as the target receptor through which S1P regulates YAP signaling, thereby promoting hepatocyte proliferation. Subsequent studies demonstrated that elevated synthesis of S1P in macrophages induced by MSCs treatment not only conferred resistance against necroptosis but also facilitated hepatocyte proliferation through delivery. This indicates that MSCs have a multifaceted role in modulating macrophages for AS‐AIH treatment. Additionally, we revealed that S1P, which promotes liver regeneration during AS‐AIH, is primarily produced by the hepatocytes themselves. The SP1/SK1 axis plays a crucial role during this period, and MSCs targeting this pathway can directly regulate hepatocyte proliferation.

This study revealed that inflammatory macrophage necroptosis is the key pathogenesis underlying AS‐AIH and identified the core mechanism that initiates liver regeneration after injury. These are closely related to the SP1/SK1/S1P axis in target cells. By delivering SP1, MSCs can alleviate inflammatory liver damage and promote regeneration. This provides a theoretical foundation for using MSCs as a potential therapeutic modality for AS‐AIH.

## 4 Experimental Section

Detailed experimental methods are provided in Supporting Information.

## Conflict of Interest

The authors declare no conflict of interest.

## Author Contributions

The study was designed by J.W. and H.R., with experiments, data acquisition, and analyses conducted by R.A., Z.Z., and J.W.; manuscript preparation was handled by R.A., Y.C., J.W., and H.R., while critical review and comments were provided by W.G., J.W., and H.R.

## Supporting information



Supporting Information

## Data Availability

The data that support the findings of this study are available on request from the corresponding author. The data are not publicly available due to privacy or ethical restrictions.
