# Supplementary material for: MSCs Suppress Macrophage Necroptosis and Foster Liver Regeneration by Modulating SP1/SK1 Axis in Treating Acute Severe Autoimmune Hepatitis
Source: Adv Sci (Weinh). 2025 Feb 3;12(12):2408974. doi: 10.1002/advs.202408974 (PMC11948073; doi:10.1002/advs.202408974)
Supplement: Supplementary file 1 — Supporting Information [file ADVS-12-2408974-s001.docx]

**MSCs suppress macrophage necroptosis and foster liver regeneration by modulating SP1/SK1 axis in treating acute severe autoimmune hepatitis**

Ran An, Zhengyi Zhu, Yuyan Chen, Wenxian Guan*, Jinglin Wang*, Haozhen Ren*

***Correspondence:** Wenxian Guan (15850502391@163.com)

Jinglin Wang (cw20120817@163.com)

Haozhen Ren (renhaozhen1984@163.com)

Table of contents

Supplementary materials and methods............................................................2

Supplementary figures....................................................................................14

Supplementary tables.....................................................................................27

Supplementary references..............................................................................31

**Supplementary materials and methods**

**Animals and treatments**

Male C57BL/6 mice, aged 4-6 and 6-8 weeks, were obtained from Animal Center of the Affiliated Drum Tower Hospital of Nanjing University Medical School. All mice were housed in specific pathogen-free facilities. All animal experiments were carried out with the approval of the Committee on the Ethics of Animal Experiments of the Affiliated Drum Tower Hospital of Nanjing University Medical School (Approval No. 2024AE01031), and were conducted in accordance with the National Institutes of Health Guidelines for the Protection and Use of Laboratory Animals.

Given that 10-20 mg kg^-1^ is the common dose of concanavalin A (ConA) used to induce autoimmune hepatitis (AIH) model in mice.^[1-4]^ 30 mg kg^-1^ ConA (C2010, Sigma-Aldrich, St. Louis, MO, USA) was administered through the tail vein injection to establish a mouse model of acute severe autoimmune hepatitis (AS-AIH). To investigate the primary modes of cell death in AS-AIH, 6 mg kg^-1^ of Nec-1s (HY-14622A, MedChemExpress, Shanghai, China) was administered via tail vein injection 30 min before ConA administration, or 20 mg kg^-1^ of Z-VAD (HY-16658B, MedChemExpress, Shanghai, China) was administered intraperitoneally. Additionally, 9.5 mg kg^-1^ of UAMC-3203 (HY-112909, MedChemExpress, Shanghai, China) was administered intraperitoneally 1 h prior, and 100 mg kg^-1^ of VX-765 (HY-13205, MedChemExpress, Shanghai, China) was administered intraperitoneally 2 hours before ConA. Additionally, 2 × 10^6^ mesenchymal stem cells (MSCs) were administered via tail vein injection 1 h after ConA administration to evaluate their therapeutic efficacy. To investigate the involvement of the specificity protein 1/ sphingosine kinase 1 (SP1/SK1) signaling pathway in MSCs treatment of AS-AIH, SP1 inhibitor, mithramycin A (MrA) (HY-A0122, MedChemExpress) was administered at a dose of 100 μg kg^-1^ via tail vein injection or SK1-specific inhibitor, PF-543 (HY-15425, MedChemExpress) was injected into the abdominal cavity at a dose of 10 mg kg^-1^ half an hour before the use of MSCs. Replacement therapy was conducted using MSCs with lentivirus-mediated (GeneChem, Shanghai, China) transfection of specific short hairpin knockdown of SP1 (shSP1-MSCs). Samples were collected 12 h or 36 h after modeling for subsequent experiments.

To ascertain whether sphingosine-1-phosphate (S1P) modulates yes-associated protein (YAP) signaling in hepatocytes by binding to S1P receptor 2 (S1PR2) during liver injury, JTE-013 (HY-100675, MedChemExpress) was administered at a dose of 20 mg kg^-1^ intraperitoneally 1 h prior to ConA treatment. Samples were collected 36 h after modeling for subsequent experiments.

*S1pr2*-cko mice were generated by crossing *S1pr2* ^fl/fl^ mice with mice carrying the Cre recombinase gene under the control of the *Alb* promoter. Mice were categorized into two groups: control mice (*S1pr2* ^fl/fl^) and conditional knockout mice (*S1pr2* ^fl/fl^; *Alb*-Cre). The AS-AIH model was established in these two types of mice using ConA, followed by treatment with MSCs to investigate the role of the S1P/S1PR2/YAP axis in liver regeneration.

**Isolation of primary cells**

MSCs were isolated and identified from bone marrow of 4- to 6-week-old C57BL/6 mice using previously described methods.^[5]^ Briefly, The surrounding soft tissues of femur, and tibia were detached and then medullary cavities were exposed. Bone marrow cells were obtained by flushing the marrow cavity with sterile phosphate buffered saline (PBS) and then centrifuging the collected fluid at 1200 rpm for 5 min. Cells were finally resuspended and cultured in low-glucose (1 g L^-1^) Dulbecco's Modified Eagle Medium (DMEM). The medium was replaced to eliminate non-adherent cells, and the remaining cells were passaged once reached approximately 80% confluence.

Primary hepatocytes and macrophages were obtained from 6- to 8-week-old C57BL/6 mice, following method as previously described.^[6,7]^ Briefly, livers underwent digestion through in situ portal vein perfusion, and then the treated livers were transferred to sterile dishes. The liver capsule was delicately torn to release the cells into the medium, followed by centrifugation at 50 × g for 3 min to separate parenchymal and non-parenchymal cells. Intrahepatic macrophages were identified as F4/80 positive non-parenchymal cells sorted by flow cytometry (FC).

**Cell lines**

AML12 cells were purchased from ATCC (Gaithersburg, MD, USA).

**Cell culture and treatments**

MSCs were cultured in low-glucose (1 g L^-1^) DMEM and primary macrophages were cultured in high-glucose (4.5 g L^-1^) DMEM. AML12 cells and primary hepatocytes were cultured in DMEM/F12 mixed with cell maintenance supplement pack (CM4000, Sigma-Aldrich). All mediums were supplemented with 10% FBS (26010074, Gibco, Grand Island, NY, USA), as well as penicillin and streptomycin (100 U mL^-1^, 100 μg mL^-1^, 15070063, Gibco), and were incubated at 37 °C in a humidified atmosphere with 5% CO2.

Co-culture system was employed to assess the impact of MSCs on macrophages. Briefly, macrophages seeded at a density of 1 × 10^6^ cells per well in lower chamber of six-well plate (3335, Corning, NY, USA) and 5 × 10^5^ MSCs were seeded in upper chamber (0.4 μm pore membrane, 3412, Corning), serving as the treatment group. To establish the model of necroptosis, macrophages were pretreated with Z-VAD (50 × 10^-6^ M) (HY-16658B, MedChemExpress) and SM164 (1 × 10^-6^ M) (HY-15989, MedChemExpress) for 1 h, followed by the further treatment with 100 ng mL^-1^ IFN-γ and 20 ng mL^-1^ TNF-α (315-05, 315-01A, Peprotech, NJ, USA) treatment for a duration of 12 h. To mimic in vivo experiments, treatment group was supplemented with the addition of MrA (0.5 × 10^-6^ M) or PF-543 (1 × 10^-6^ M) to medium, respectively. Replacement therapy was conducted using shSP1-MSCs. M1 polarization model for macrophages was established by the stimulation with lipopolysaccharide (500 ng mL^-1^, HY-D1056, MedChemExpress) for a duration of 24 h.

To verify the impact of S1PR2 inhibition on YAP signaling in hepatocyte, JTE-013 (10 × 10^-6^ M, MedChemExpress) was introduced into AML12 medium and incubated for 24 h.

To assess the impact of macrophages on hepatocyte proliferation, the concentrated conditioned medium of macrophage was introduced into AML12 medium. Briefly, once the in vitro model of macrophage necroptosis and co-culture treatment was established, the upper chamber and medium were discarded, and fresh medium was added to sustain the culture. After 24 h, conditioned medium was gathered and concentrated thirtyfold (4500 × g, 10 min) using centrifugal filter (UFC9003, Millipore, MA, USA). AML12 cells at a density of 5 × 10^5^ were seeded into six-well plates. Subsequently, 200 μL of the concentrate was added to medium in each well, and cells were cultured for 24 h.

**Western blotting (WB)**

WB was performed as previously described.^[8]^ Briefly, total protein of cells was extracted utilizing lysis buffer (P0013J, Beyotime, Shanghai, China), and protein concentration was assessed using bicinchoninic acid assay kit (P0012, Beyotime). Protein samples were separated on 10% sodium dodecyl sulfate-polyacrylamide gels (PG112, Epizyme, Shanghai, China) and subsequently transferred to a polyvinylidene difluoride membrane (ISEQ00010, Millipore). Protein bands were blocked with 10% skim milk and then incubated with appropriate primary and secondary antibodies. Subsequently, target proteins were visualized using a chemiluminescence gel imaging system (5200 Multi, Tanon, Shanghai, China) and analyzed using Image J software (MD, USA). Antibodies utilized for WB are detailed in ***Table S1***.

**Quantitative real-time polymerase chain reaction (qRT-PCR)**

Total RNA of cells was extracted using Monzol™ reagent (MI20201S, monadbiotech, Suzhou, China) and subjected to qRT-PCR, following previously described methods.^[8]^ Briefly, Complementary DNA (cDNA) was synthesized using cDNA Synthesis SuperMix (11141ES60, yeasen, Shanghai, China) and the concentration was measured using NanoDrop ND-1000 (NanoDrop, DE, USA). The mRNA levels of target genes were quantified using ChamQ SYBR Color qPCR Master Mix (Q431-02, vazyme, Nanjing, China) on an ViiA™ 7 Real-time fluorescence quantitative PCR system manufactured by Applied Biosystems (CA, USA). The primers utilized for qRT-PCR are listed in ***Table S2***.

**Lentivirus transfection**

Lentivirus (hU6-MCS-CBh-gcGFP-IRES-puromycin) carrying short hairpin RNA targeting SP1 (target sequence is listed in ***Table S3***) was purchased from GeneChem. MSCs were transfected with lentivirus using HitransG A (GeneChem) based on manufacturer’s protocols.

**FC analysis**

Annexin V-FITC Apoptosis Detection Kit (C1062L, Beyotime) was used to detect the proportion of apoptosis and necroptosis cells according to manufacturer’s instructions. Concerning the examination and categorization of target cells, incubation processes were conducted using antibody according to manufacturer's protocols. Briefly, cells were pre-incubated with purified anti-CD16/CD32 antibody (101302, Biolegend, CA, USA) for 10 min to minimize nonspecific binding, followed by incubation with the corresponding antibodies (target antibodies are listed in ***Table S1***). Results were gathered using flow cytometer (BD Accuri™ C6 Plus/FACSAria™ II, BD Biosciences, NJ, USA) and analyzed utilizing FlowJo 10.4 software (OR, USA).

**Histology and immunohistochemical staining**

After fixation using 4% paraformaldehyde (P0099, Beyotime) solution and embedding in paraffin, the liver tissue was cut into 5 μm slices, followed by hematoxylin-eosin staining to assess the degree of liver injury, and immunohistochemical staining to detect the expression of Ki67 in hepatocytes using previously described methods.^[8]^ At least three randomly selected areas per section were photographed to facilitate analysis. Leica DMi8 microscope (Leica, Wetzlar, Germany) was utilized for capturing images, and Image J software was employed for semi-quantitative analysis.

**Immunofluorescence (IF) staining**

Polarization and necroptosis of macrophages, along with the expression of YAP in AML12 cells were characterized by IF. Briefly, cells and tissue sections were initially fixed with 4% paraformaldehyde for 30 min, followed by a subsequent incubation with Triton X-100 (P0096, Beyotime) for 10 min. After blocking with 5% bovine serum albumin (A1933, Sigma-Aldrich) for 1h at room temperature (RT), cells or tissue sections were incubated with the primary antibody at 4 °C overnight, and subsequently, exposed to the corresponding species of secondary antibody at RT for 1h. After labeling the nucleus with DAPI (P0131, Beyotime), the expression of target proteins in cells or sections were visualized using fluorescence microscopy (Leica DMi8). TUNEL cell apoptosis detection kit was employed to detect hepatocyte apoptosis in tissue sections, and EdU Cell Proliferation Kit Alexa Fluor 555 was used to detect the proliferation of AML12 cells based on manufacturer’s protocols (C1088, C0075S, Beyotime).

**Enzyme-linked immunosorbent assay (ELISA)**

ALT, AST, TNF-α, IL-6, IL-10, and S1P concentrations in serum, tissue homogenates, cell lysates, or medium supernatant were quantified via ELISA following manufacturer's instructions (mIC50536-1, IC50325-1, mIC50274-1, ml062988, Mlbio, China).

**Chromatin immunoprecipitation (ChIP) assay**

The binding of SP1 to the promoter region of macrophage SK1 gene was evaluated utilizing BeyoChIP™ Enzymatic ChIP Assay Kit (P2083S, Beyotime). Briefly, isolated intrahepatic macrophages were crosslinked with 1% formaldehyde in PBS at 37°C for 10 min. Subsequently, chromatin extraction was carried out according to manufacturer's protocols. Following chromatin preparation, samples were subjected to immunoprecipitation at 4 °C overnight using IgG or SP1 antibodies to capture chromatin fragments. DNA fragments captured during immunoprecipitation were employed for qRT-PCR. Primers for qRT-PCR are listed in ***Table S2***.

**Luciferase reporter assay**

The promoter sequence of mouse *Sk1* (2kb upstream of transcription start site) was predicted and cloned into the luciferase reporter vector GPL4-basic. The plasmid was transfected into macrophages using Lipo8000™ Transfection Reagent (C0533, Beyotime) following manufacturer's protocols. To homogenize firefly luciferase activity, GPL4-RL expressing renilla luciferase was cotransfected. Luciferase activity was performed with a Dual Luciferase Reporter Gene Assay Kit (RG027, Beyotime) based on manufacturer’s protocols.

**Cell Counting Kit-8 (CCK-8) assay**

The viability of AML12 cells in each group was assessed using the CCK-8 kit (C0038, Beyotime) in accordance with manufacturer's guidelines. Following the completion of the reaction, the absorbance at 450 nm was quantified using microplate reader (Tecan, Switzerland).

**RNA** **sequencing (RNA-seq)**

Total RNA was extracted and purified utilizing TRIzol reagent (15596018CN, invitrogen, CA, USA) following the manufacturer's protocols. RNA concentration and purity as well as integrity were measured using NanoDrop ND-1000 and Bioanalyzer 2100 (Agilent, CA, USA), respectively. Poly(A) RNA was purified twice from 1 μg of total RNA and subjected to fragmentation by treatment at 94 °C for 5-7 min following the manufacturer's protocols (Dynabeads Oligo (dT) 25-61005, Thermo Fisher, CA, USA and Magnesium RNA Fragmentation Module, e6150, NEB, MA, USA). RNA fragments were reverse transcribed into cDNA using SuperScript™ II Reverse Transcriptase (18064022, invitrogen) and then submitted for sequencing on illumina NovaSeq™ 6000 platform.

**Assay for Transposase Accessible Chromatin sequencing (ATAC-seq)**

Intrahepatic macrophages were sorted by FC and subsequently lysed using lysis buffer (10 × 10^-3^ M Tris-HCl pH 7.4, 3 × 10^-3^ M MgCl2, 10 × 10^-3^ M NaCl, 0.1% IGEPAL CA-630). The nuclei were treated with DNA library prep kit (TD501, Vazyme) to prepare libraries. Subsequently, the prepared libraries underwent purification using VAHTS DNA Clean Beads (N41101, Vazyme) and then analyzed using 2100 Bioanalyzer. Finally, the libraries were utilized for high-throughput sequencing on illumina NovaSeq™ 6000 platform.

**Cleavage Under Targets & Tagmentation (****CUT&Tag)**

Intrahepatic macrophages were sorted by FC and subsequently incubated with ConA coated magnetic beads for 15 min at RT. Then, bead-bound cells were resuspended and incubated with SP1 antibody (21962-1-AP, proteintech, Wuhan, China) overnight at 4°C. After incubation, secondary antibody (ab6702, abcam) was added to this mixture for 30 min at RT. Afterward, the pA-Tn5 adapter complex were introduced into the sample and allowed to react for 1 h at RT. Following this, samples underwent tagmentation at 37°C for 1 h to achieve DNA fragmentation. Libraries were constructed from the immunoprecipitated DNA using NEBNext HiFi 2XPCR Master Mix (NEB, MA, USA) and then purified using VAHTS DNA Clean Beads. Finally, the libraries were utilized for high-throughput sequencing on illumina NovaSeq™ 6000 platform.

**Statistical analysis**

Data analysis was conducted using GraphPad Prism 8.0 (GraphPad Software, CA, USA). The survival curve was generated using the Kaplan-Meier method with log-rank test. All experiments were repeated a minimum of three times, and data are presented as mean±standard error of the mean. Statistical comparisons between two groups were performed using Student’s t-test. For comparisons among multiple groups, one-way ANOVA was employed, followed by Tukey's post hoc test. Statistical significance was considered at *p*<0.05 (n.s.=not significant, **p* <0.05, ***p* <0.01, ****p* <0.001 and *****p* <0.0001).

**
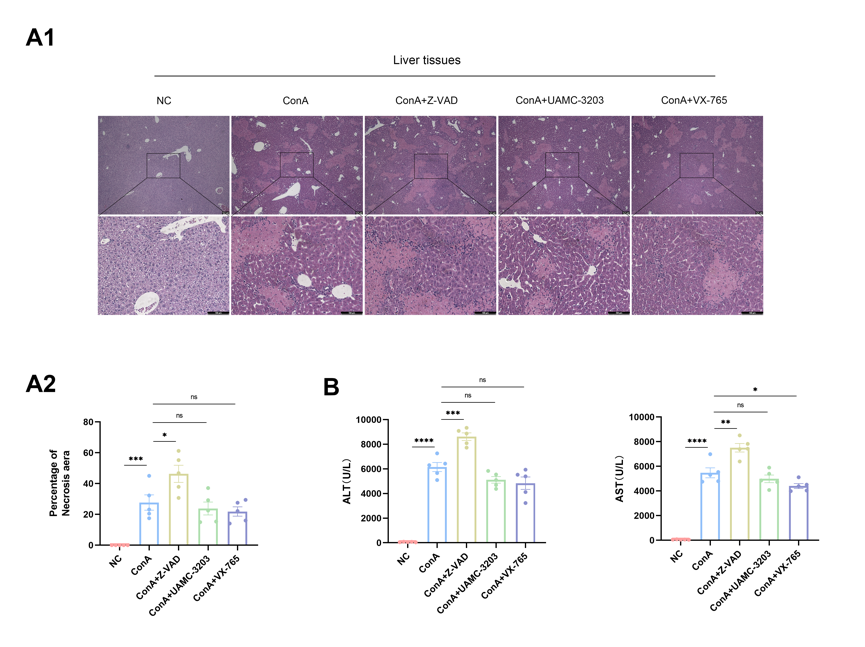
**

**Figure S1.** Necroptosis is the predominant mode of cell death in AS-AIH. A) Representative H&E staining images showed the histological morphology of liver tissues (scale bars, 100 μm) (n=5). B) The levels of ALT and AST in serum were detected by ELISA (n=5). Data are presented as means ± SEM, ^∗^*p* <0.05, ^∗∗^*p* <0.01, ^∗∗∗^*p* <0.001, ^∗∗∗∗^*p* <0.0001 by Student’s t test.

**
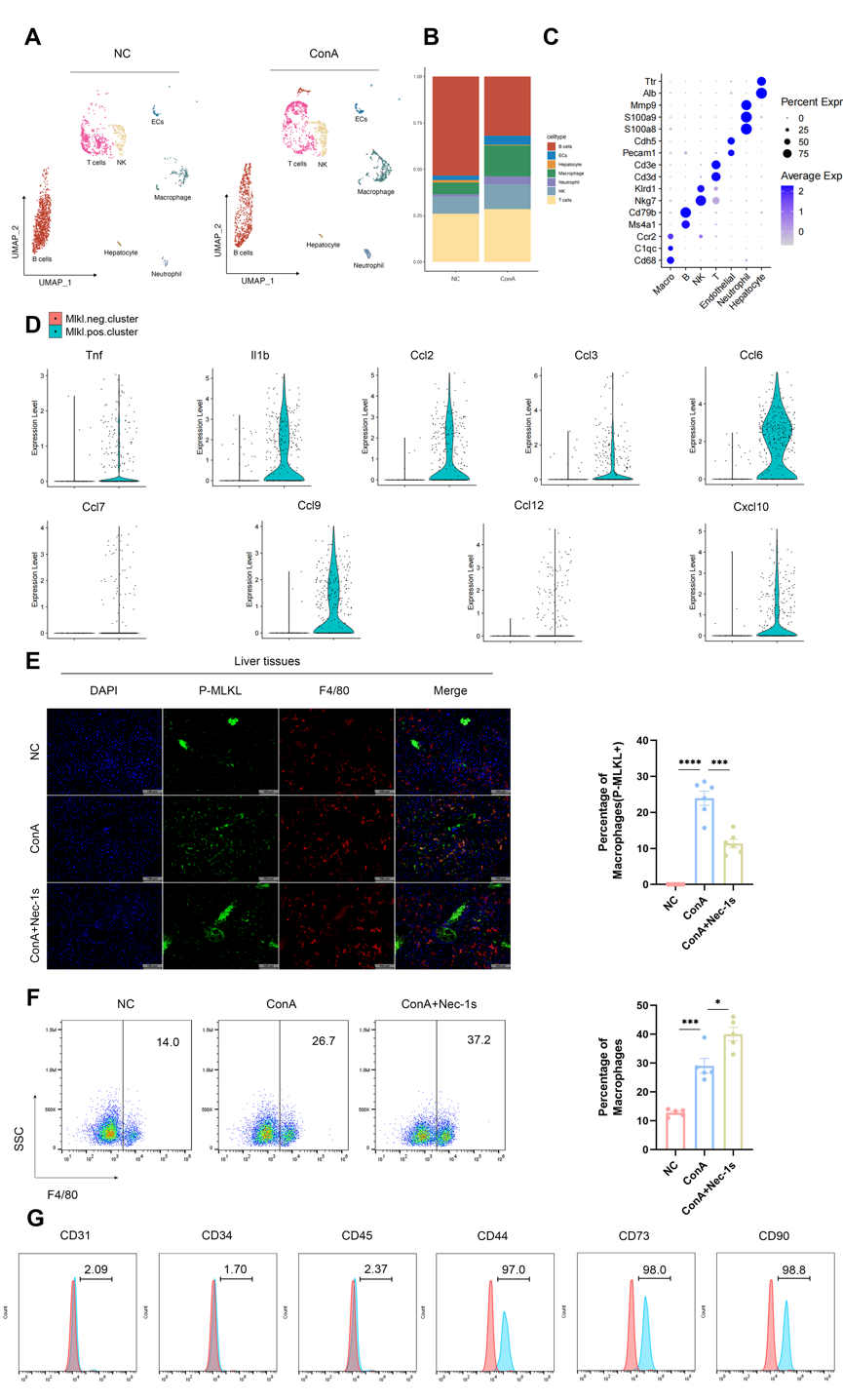
**

**Figure S2.** Macrophage necroptosis is involved in the progression of AS-AIH. A) UMAP visualisation of liver cell subsets in NC and ConA groups, annotated and colored by clustering. B) The proportion of each cell cluster within the liver of two groups. C) Representative genes for each cluster. D) Expression of representative inflammatory factors and chemokines in two distinct macrophage clusters. E) Representative IF images showed the expression of P-MLKL in intrahepatic macrophages (scale bars, 100 μm) (n=6). F) Percentage of macrophages in liver non-parenchymal cells analyzed via FC (n=5). G) FC analysis of BMSC markers (n=3). Data are presented as means ± SEM, ^∗^*p* <0.05, ^∗∗^*p* <0.01, ^∗∗∗^*p* <0.001, ^∗∗∗∗^*p* <0.0001 by Student’s t test.


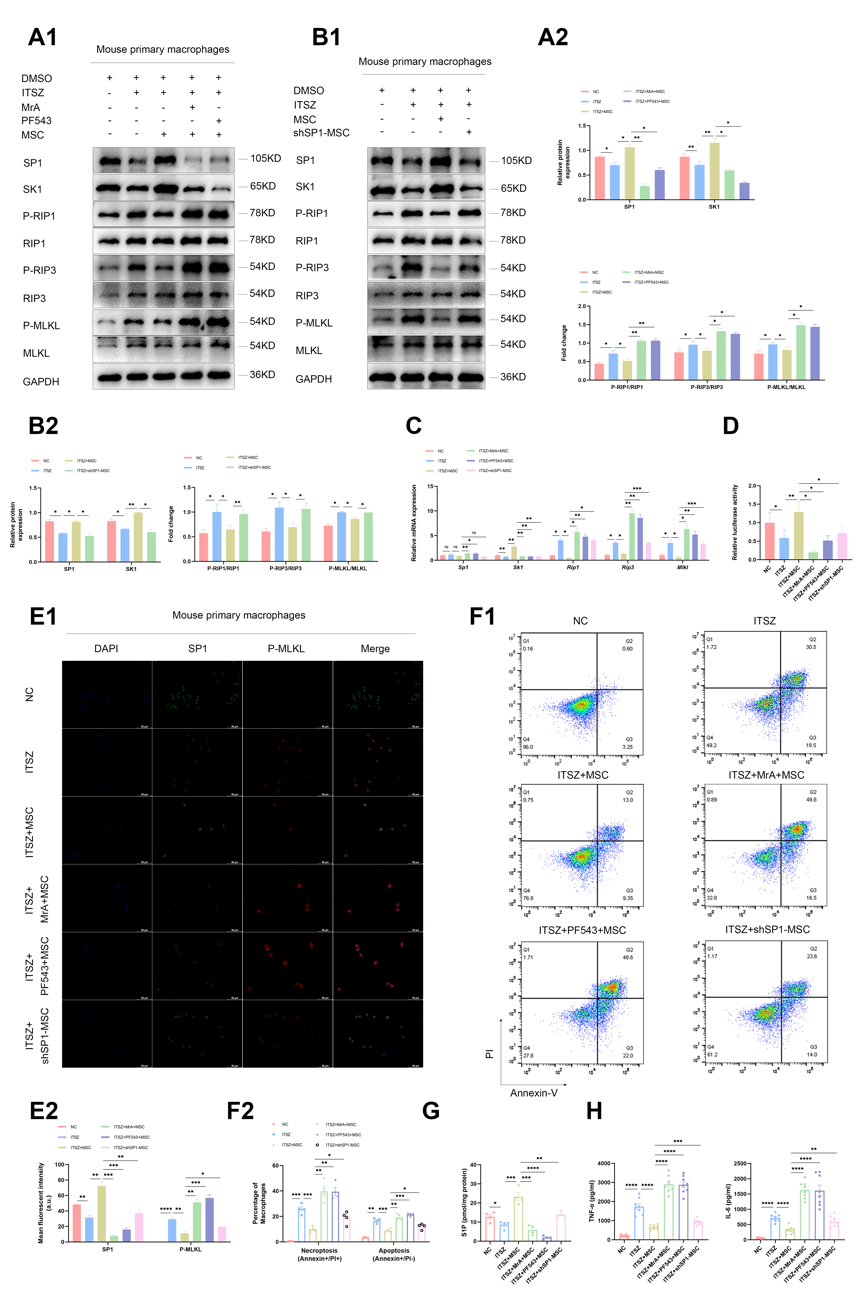


**Figure S3.** MSCs suppress macrophage necroptosis by delivering SP1 to facilitate the transcription of *Sk1*. A-C) In vitro, ITSZ were employed to induce primary mouse macrophages necroptosis, while MrA, PF543, and shSP1-MSC were utilized to validate the therapeutic efficacy of MSCs. The protein and mRNA levels of SP1/SK1 axis and necroptosis signaling pathway in macrophages were measured (n=3). D) Macrophages were co-transfected with luciferase reporter plasmid containing *Sk1* promoter sequence and renilla plasmid for 12 h, and luciferase activity was assessed 12 h after establishing necroptosis and each treatment model (n=3). E) Representative IF images showed the expression of SP1 and P-MLKL in mouse primary macrophages (scale bars, 50 μm) (n=3). F) FC analysis of necrotic and apoptotic proportion in mouse primary macrophages (n=4). G) S1P concentrations in mouse primary macrophages were detected by ELISA (n=4). H) The concentrations of TNF-α and IL-6 in macrophage supernatants were detected by ELISA (n=8). Data are presented as means ± SEM, ^∗^*p* <0.05, ^∗∗^*p* <0.01, ^∗∗∗^*p* <0.001, ^∗∗∗∗^*p* <0.0001 by Student’s t test.


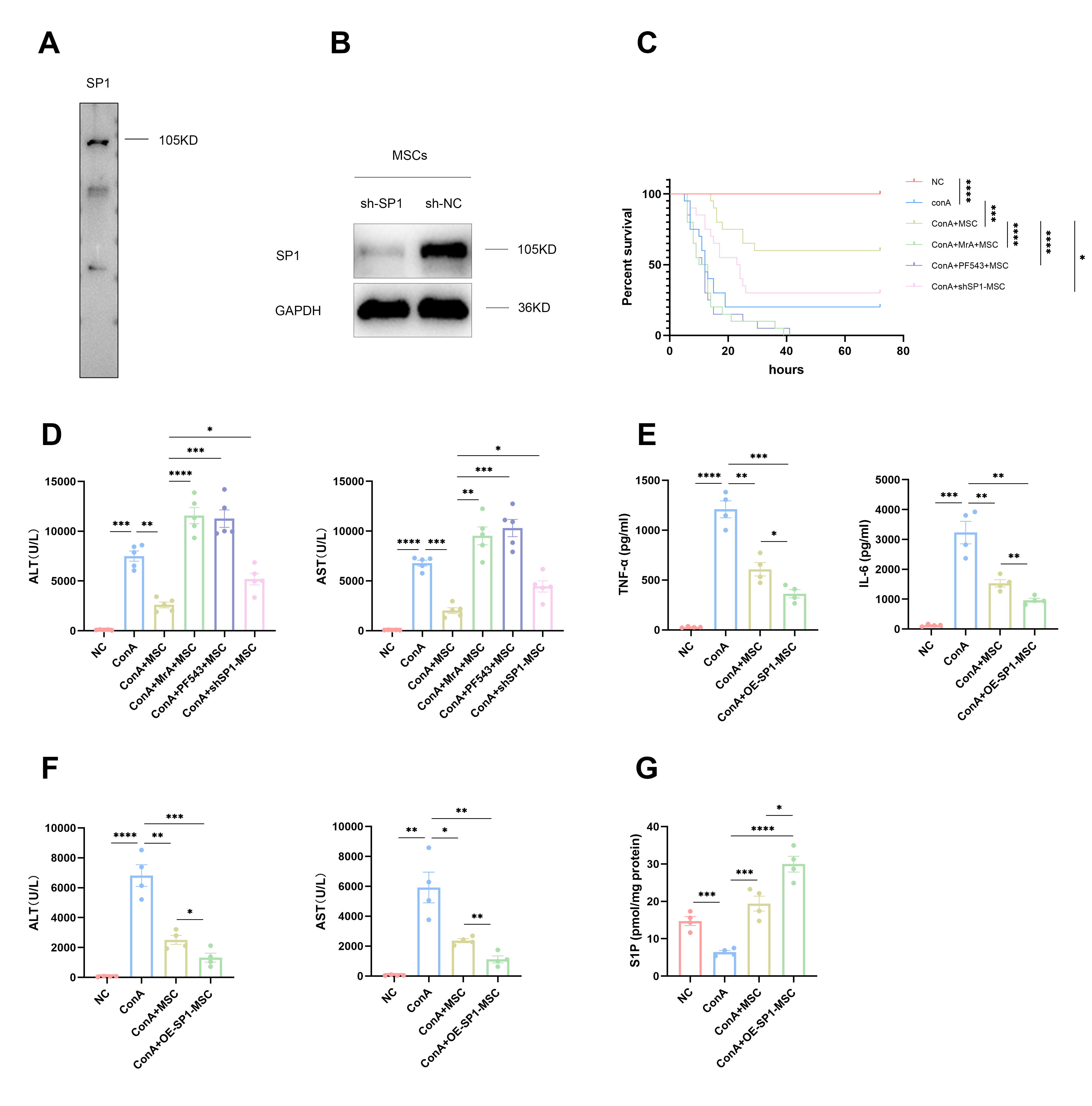


**Figure S4.** MSCs exert a therapeutic role in AS-AIH by delivering SP1. A) WB analysis revealed the presence of SP1 in the supernatant of MSCs. B) WB analysis demonstrated successful transfection of MSCs with lentivirus containing specific short hairpin RNA targeting SP1. C) Kaplan-Meier survival curves of mice in each experimental group (n=20). D) Serum levels of ALT and AST were detected by ELISA (n=5). E) Serum levels of TNF-a and IL-6 were detected by ELISA (n=4). F) Serum levels of ALT and AST were detected by ELISA (n=4). G) S1P concentrations in intrahepatic macrophages were detected by ELISA (n=4). Data are presented as means ± SEM, ^∗^*p* <0.05, ^∗∗^*p* <0.01, ^∗∗∗^*p* <0.001, ^∗∗∗∗^*p* <0.0001 by Student’s t test and Log-rank test.


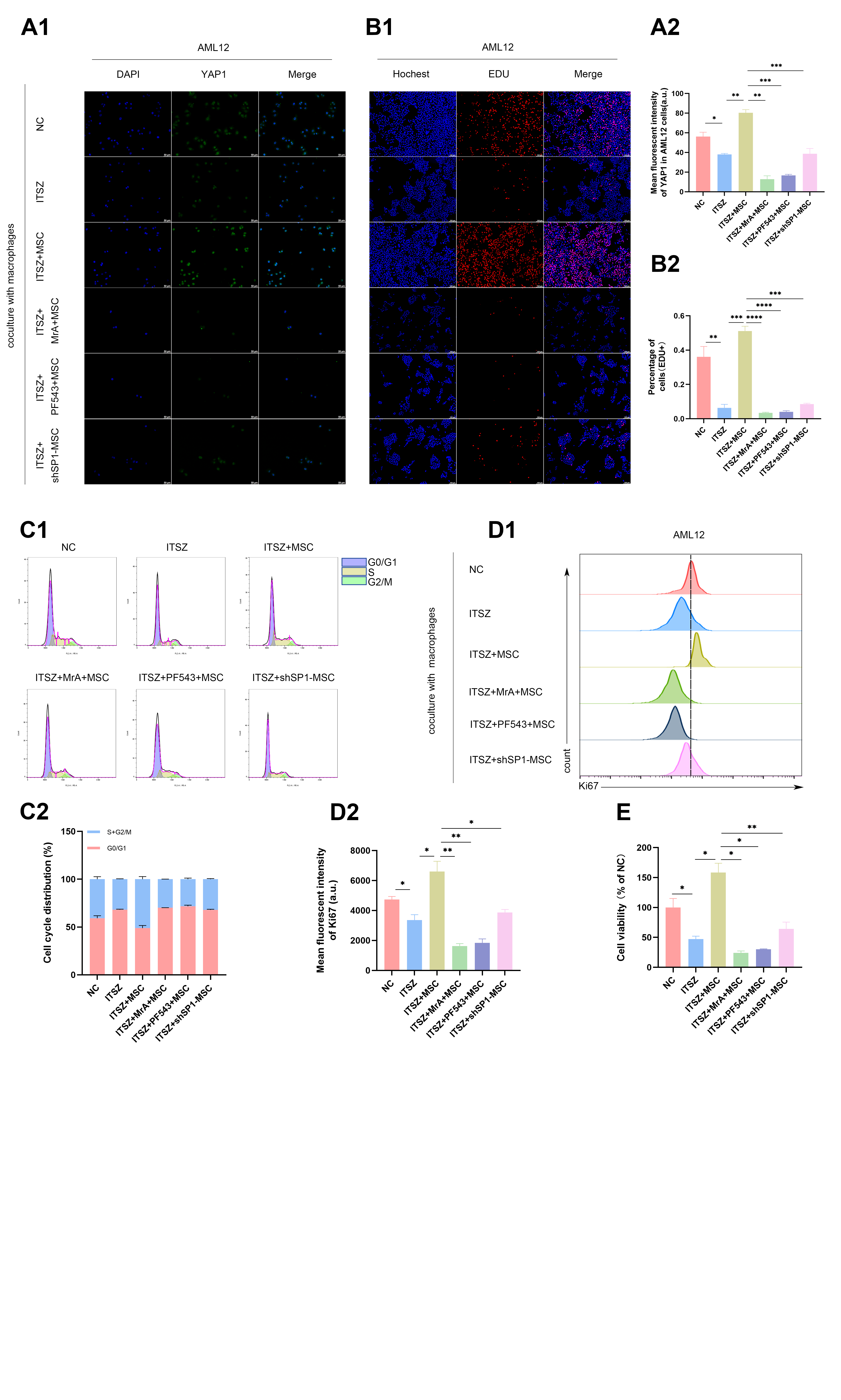


**Figure S5.** MSCs indirectly regulate Hippo/YAP signaling pathway in hepatocytes to promote liver regeneration by enhancing S1P synthesis in macrophages. A) Representative IF images showed the expression of YAP1 in AML12 cells (scale bars, 50 μm) (n=3). B) The EdU assay was conducted to examine the cell proliferation of AML12 under various treatment conditions (scale bars, 100 μm) (n=3). C) FC analysis of AML12 cells proportion in different cell cycles (n=3). D) FC analysis of Ki67 expression in AML12 cells (n=3). E) CCK8 assay was employed to assess the viability of AML12 cells (n=3). Data are presented as means ± SEM, ^∗^*p* <0.05, ^∗∗^*p* <0.01, ^∗∗∗^*p* <0.001, ^∗∗∗∗^*p* <0.0001 by Student’s t test.


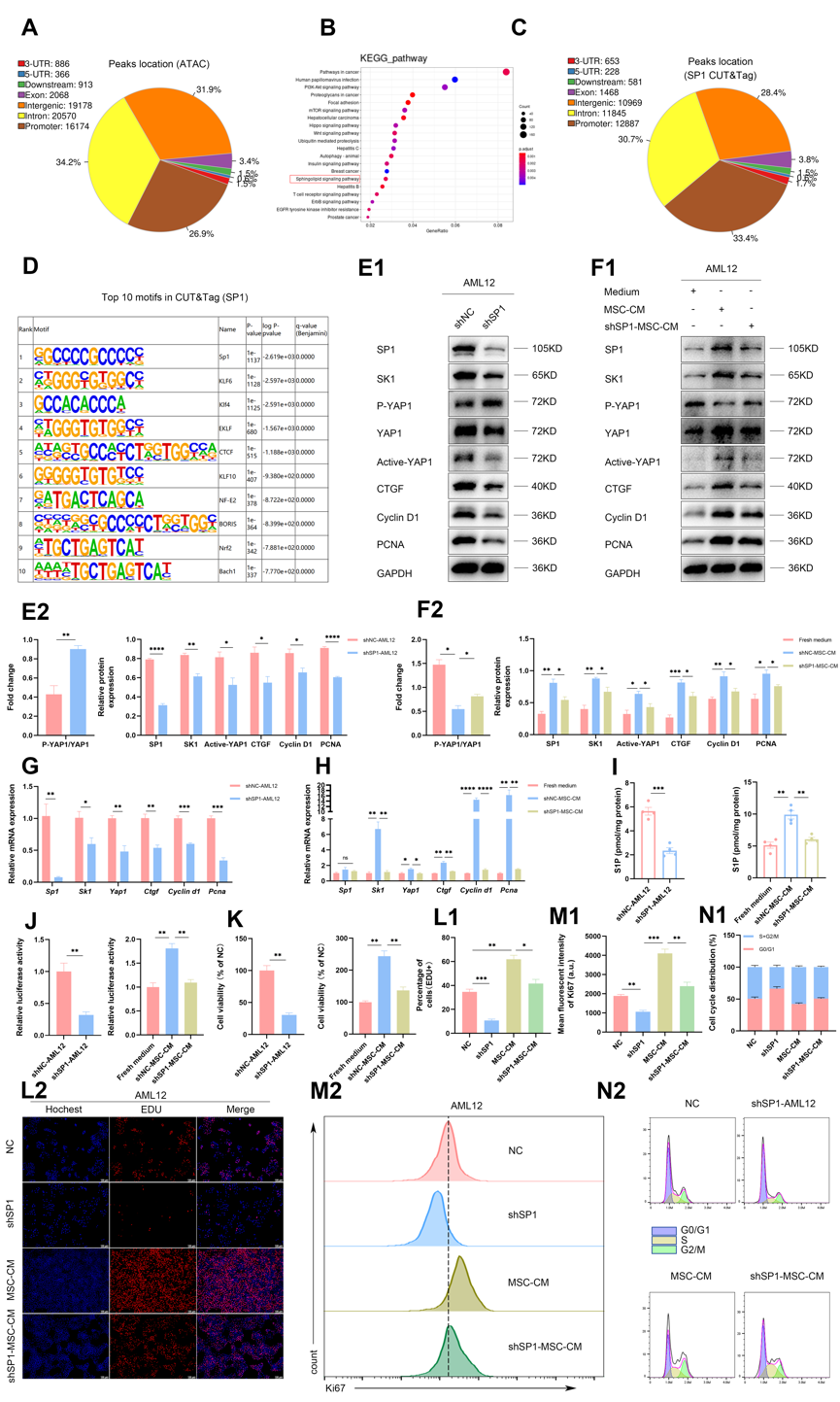


**Figure S6.** MSCs promote hepatocyte proliferation by directly regulating the SK1/S1P/YAP axis through the delivery of SP1. A) Locations of OCRs in hepatocytes identified through ATAC-seq analysis. B) KEGG enrichment analysis based on the gene ratio identified in ATAC-seq data. C) The peak distribution detected in CUT&Tag analysis of hepatocytes. D) The top 10 motifs identified in CUT&Tag analysis. E) WB analysis of the involvement of SP1 in regulating the SK1/S1P/YAP axis in AML12 cell proliferation (n=3). F) WB analysis of the impact of MSC-mediated SP1 delivery on the SK1/S1P/YAP axis in AML12 cells (n=3). G) QRT-PCR analysis of the involvement of SP1 in regulating the SK1/S1P/YAP axis in AML12 cell proliferation (n=3). H) QRT-PCR analysis of the impact of MSC-mediated SP1 delivery on the SK1/S1P/YAP axis in AML12 cells (n=3). I) S1P concentrations in AML12 cells were detected by ELISA (n=4). J) The regulatory role of SP1 in Sk1 transcription in AML12 cells and the therapeutic efficacy of MSC-mediated SP1 delivery were confirmed through luciferase activity measurements (n=3). K) CCK8 assay was employed to assess the viability of AML12 cells (n=3). L) The EdU assay was conducted to examine the cell proliferation of AML12 under various treatment conditions (scale bars, 100 μm) (n=3). M) FC analysis of Ki67 expression in AML12 cells (n=3). N) FC analysis of AML12 cells proportion in different cell cycles (n=3). Data are presented as means ± SEM, ^∗^*p* <0.05, ^∗∗^*p* <0.01, ^∗∗∗^*p* <0.001, ^∗∗∗∗^*p* <0.0001 by Student’s t test.

**
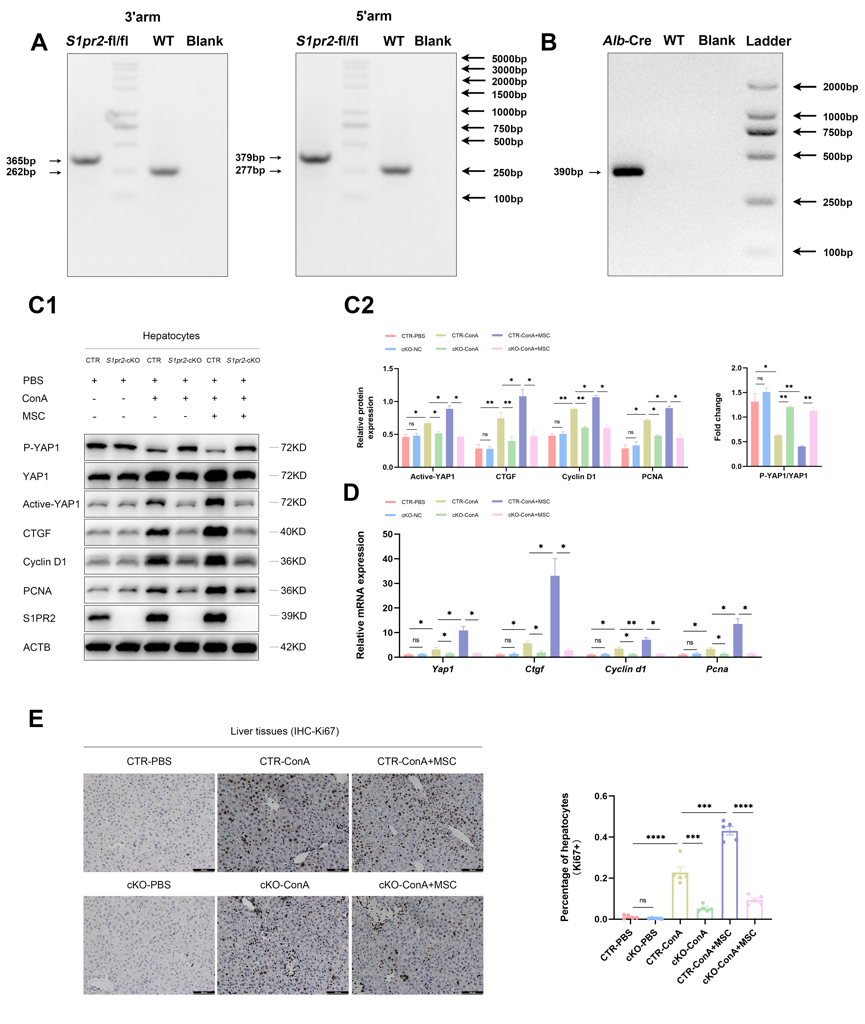
**

**Figure S7.** The S1P/S1PR2/YAP axis is the key signaling pathway regulating hepatocyte proliferation during AS-AIH. A) Identification of the flox sequences inserted into the *S1pr2* gene (*S1pr2*^fl/fl^). B) Identification of the *Alb*-cre genotype. C) WB analysis of YAP signaling pathway in hepatocytes in vivo (n=3). D) The mRNA levels of *Yap1*, *Ctgf*, *Cyclin d1* and *Pcna* in hepatocytes in vivo (n=3). E) Representative IHC images showed the Ki67-positive hepatocytes proportion in liver tissues (scale bars, 100 μm) (n=5). Data are presented as means ± SEM, ^∗^*p* <0.05, ^∗∗^*p* <0.01, ^∗∗∗^*p* <0.001, ^∗∗∗∗^*p* <0.0001 by Student’s t test.

**Table S1: Antibodies**

| **Name** | **Supplier** | **Cat no.** | **Clone no.** |
| --- | --- | --- | --- |
| Anti-SP1 | Proteintech | 21962-1-AP | Polyclonal |
| Anti-SP1 | Proteintech | 66508-1-Ig | 4G3H3 |
| Anti-SPHK1 | ABclonal | A0660 | Polyclonal |
| Anti-RIP | Cell Signaling Technology | #3493 | D94C12 |
| Anti-Phospho-RIPK1 (Ser166) | Affinity | #AF2398 | Polyclonal |
| Anti-RIP3 | Proteintech | 17563-1-AP | Polyclonal |
| Anti-RIP3 (phospho T231 + S232) | Abcam | ab222320 | EPR19403-52 |
| Anti-MLKL | Proteintech | 66675-1-Ig | 3D4C6 |
| Anti-Phospho-MLKL (Ser345) | Cell Signaling Technology | #37333 | D6E3G |
| Anti-YAP | Cell Signaling Technology | #14074 | D8H1X |
| Anti-Active YAP1 | Abcam | ab205270 | EPR19812 |
| Anti-Phospho-YAP (Ser127) | Cell Signaling Technology | #13008 | D9W2I |
| Anti-CTGF | Affinity | #DF7091 | Polyclonal |
| Anti-Cyclin D1 | Abcam | ab134175 | EPR2241 |
| Anti-PCNA | Abcam | ab92552 | EPR3821 |
| Anti-PCNA | Proteintech | 10205-2-AP | Polyclonal |
| Anti-F4/80 | Proteintech | 28463-1-AP | Polyclonal |
| Anti-iNOS | Abcam | ab49999 | NOS-IN |
| Anti-Mannose Receptor | Abcam | ab64693 | Polyclonal |
| Anti-Ki67 | Abcam | ab16667 | SP6 |
| Anti-IgG | Proteintech | 30000-0-AP | Polyclonal |
| Anti-GAPDH | Proteintech | 60004-1-Ig | 1E6D9 |
| Anti-Beta Actin | Proteintech | 66009-1-Ig | 2D4H5 |
| Goat anti-Rabbit IgG H&L | Abcam | ab6702 | Polyclonal |
| Goat anti-Rabbit IgG H&L (HRP) | Abcam | ab205718 | Polyclonal |
| Goat anti-Mouse IgG H&L (HRP) | Abcam | ab205719 | Polyclonal |
| Goat Anti-Rabbit IgG H&L (Alexa Fluor® 488/594) | Abcam  Abcam | ab150077  ab150080 | Polyclonal  Polyclonal |
| Goat Anti-Mouse IgG H&L (Alexa Fluor® 488/594 | Abcam  Abcam | ab150113  ab150116 | Polyclonal  Polyclonal |
| Anti-mouse CD16/32 | Biolegend | #101302 | 93 |
| Anti-mouse F4/80 | Biolegend | #157318 | QA17A29 |
| Anti-mouse Nos2 | Biolegend | #696806 | W16030C |
| Anti-mouse CD206 | Biolegend  Biolegend | #141704  #141708 | C068C2  C068C2 |
| Anti-mouse Ki-67 | Biolegend | #652424 | 16A8 |
| Anti-mouse CD31 | Biolegend | #160212 | W18222B |
| Anti-mouse CD34 | BD Biosciences | #560238 | RAM34 |
| Anti-mouse CD45 | Biolegend | #157214 | S18009F |
| Anti-mouse CD44 | Biolegend | #156008 | NIM-R8 |
| Anti-mouse CD73 | Biolegend | #127220 | TY/11.8 |
| Anti-mouse CD90 | Biolegend | #140304 | 53-2.1 |
| Anti-mouse S1PR2 | Affinity | #DF4921 | Polyclonal |

**Table S2: Primers for qRT-PCR**

| **Name** | **Sequence** | **Supplier** |
| --- | --- | --- |
| *Sp1*-F | AGGGTCCGAGTCAGTCAGG | Tsingke Biotechnology Co., Ltd. |
| *Sp1*-R | CTCGCTGCCATTGGTACTGTT | Tsingke Biotechnology Co., Ltd. |
| *Sk1*-F | GGTGAATGGGCTAATGGAACG | Tsingke Biotechnology Co., Ltd. |
| *Sk1*-R | CTGCTCGTACCCAGCATAGTG | Tsingke Biotechnology Co., Ltd. |
| *Ripk1*-F | GACAGACCTAGACAGCGGAG | Tsingke Biotechnology Co., Ltd. |
| *Ripk1*-R | CCAGTAGCTTCACCACTCGAC | Tsingke Biotechnology Co., Ltd. |
| *Ripk3*-F | CAGTGGGACTTCGTGTCCG | Tsingke Biotechnology Co., Ltd. |
| *Ripk3*-R | CAAGCTGTGTAGGTAGCACATC | Tsingke Biotechnology Co., Ltd. |
| *Mlkl*-F | TTAGGCCAGCTCATCTATGAACA | Tsingke Biotechnology Co., Ltd. |
| *Mlkl*-R | TGCACACGGTTTCCTAGACG | Tsingke Biotechnology Co., Ltd. |
| *Inos*-F | GTTCTCAGCCCAACAATACAAGA | Tsingke Biotechnology Co., Ltd. |
| *Inos*-R | GTGGACGGGTCGATGTCAC | Tsingke Biotechnology Co., Ltd. |
| *Cd206*-F | CTCTGTTCAGCTATTGGACGC | Tsingke Biotechnology Co., Ltd. |
| *Cd206*-R | TGGCACTCCCAAACATAATTTGA | Tsingke Biotechnology Co., Ltd. |
| *Tnfα*-F | CAGGCGGTGCCTATGTCTC | Tsingke Biotechnology Co., Ltd. |
| *Tnfα*-R | CGATCACCCCGAAGTTCAGTAG | Tsingke Biotechnology Co., Ltd. |
| *Il6*-F | CTGCAAGAGACTTCCATCCAG | Tsingke Biotechnology Co., Ltd. |
| *Il6*-R | AGTGGTATAGACAGGTCTGTTGG | Tsingke Biotechnology Co., Ltd. |
| *Il10*-F | CTTACTGACTGGCATGAGGATCA | Tsingke Biotechnology Co., Ltd. |
| *Il10*-R | GCAGCTCTAGGAGCATGTGG | Tsingke Biotechnology Co., Ltd. |
| *S1pr1*-F | ATGGTGTCCACTAGCATCCC | Tsingke Biotechnology Co., Ltd. |
| *S1pr1*-R | CGATGTTCAACTTGCCTGTGTAG | Tsingke Biotechnology Co., Ltd. |
| *S1pr2*-F | ACAGCAAGTTCCACTCAGCAA | Tsingke Biotechnology Co., Ltd. |
| *S1pr2*-R | CTGCACGGGAGTTAAGGACAG | Tsingke Biotechnology Co., Ltd. |
| *S1pr3*-F | ACTCTCCGGGAACATTACGAT | Tsingke Biotechnology Co., Ltd. |
| *S1pr3*-R | CCAAGACGATGAAGCTACAGG | Tsingke Biotechnology Co., Ltd. |
| *Yap1*-F | ACCCTCGTTTTGCCATGAAC | Tsingke Biotechnology Co., Ltd. |
| *Yap1*-R | TGTGCTGGGATTGATATTCCGTA | Tsingke Biotechnology Co., Ltd. |
| *Ctgf*-F | GGCCTCTTCTGCGATTTCG | Tsingke Biotechnology Co., Ltd. |
| *Ctgf*-R | GCAGCTTGACCCTTCTCGG | Tsingke Biotechnology Co., Ltd. |
| *Cyclin d1*-F | GCGTACCCTGACACCAATCTC | Tsingke Biotechnology Co., Ltd. |
| *Cyclin d1*-R | ACTTGAAGTAAGATACGGAGGGC | Tsingke Biotechnology Co., Ltd. |
| *Pcna*-F | TTGCACGTATATGCCGAGACC | Tsingke Biotechnology Co., Ltd. |
| *Pcna*-R | GGTGAACAGGCTCATTCATCTCT | Tsingke Biotechnology Co., Ltd. |
| *Beta-actin*-F | GTGACGTTGACATCCGTAAAGA | Tsingke Biotechnology Co., Ltd. |
| *Beta-actin*-R | GCCGGACTCATCGTACTCC | Tsingke Biotechnology Co., Ltd. |
| *Gapdh*-F | AGGTCGGTGTGAACGGATTTG | Tsingke Biotechnology Co., Ltd. |
| *Gapdh*-R | TGTAGACCATGTAGTTGAGGTCA | Tsingke Biotechnology Co., Ltd. |
| *Sk1*-promoter-CHIP-F  （macrophage） | CGGGAATGGACAAGAACA | Tsingke Biotechnology Co., Ltd. |
| *Sk1*-promoter-CHIP-R  （macrophage） | TGCTGACAGGCTGGCTAAT | Tsingke Biotechnology Co., Ltd. |
| *Sk1*-promoter-CHIP-F  (hepatocyte) | AGAGGTATTGCGGTGTCCCA | Tsingke Biotechnology Co., Ltd. |
| *Sk1*-promoter-CHIP-R  (hepatocyte) | GCCTTTAATCCCAGCACTCG | Tsingke Biotechnology Co., Ltd. |
| *S1pr2*-flox  (5'arm)-F1 | GCACATCCTGTGTAGTCCAGACTG | Tsingke Biotechnology Co., Ltd. |
| *S1pr2*-flox  (5'arm)-R1 | GGGATCAAAGGTGTACTAAACTATGTGA | Tsingke Biotechnology Co., Ltd. |
| *S1pr2*-flox  (3'arm)-F2 | GGATTCTATGGTGGTTGTAATTGGCC | Tsingke Biotechnology Co., Ltd. |
| *S1pr2*-flox  (3'arm)-R2 | TGAGCGCAGGTGGAAGCTAGA | Tsingke Biotechnology Co., Ltd. |
| *Alb*-Cre-F | TTGGCCCCTTACCATAACTG | Tsingke Biotechnology Co., Ltd. |
| *Alb-*Cre-R | GAAGCAGAAGCTTAGGAAGATGG | Tsingke Biotechnology Co., Ltd. |

**Table S3:** **Target sequence for short hairpin RNA**

| **shRNA** | **Target sequence** |
| --- | --- |
| SP1-shRNA | 5'-CCTTCACAACTCAAGCTATTT-3' |

**Supplementary references**

[1] H. Yuting, D. Mingjie, Z. Jiakai, H. Changjun, S. Jihua, W. Yun, T. Ruolin, W. Zeyu, G. Wenzhi, *Adv Sci (Weinh)* **2024**.

[2] Y. He, D. Feng, S. Hwang, B. Mackowiak, X. Wang, X. Xiang, R. M. Rodrigues, Y. Fu, J. Ma, T. Ren, Y. Ait-Ahmed, M. Xu, S. Liangpunsakul, B. Gao, *J Hepatol* **2021**, *75*, 163.

[3] Y. Wang, X. Guo, G. Jiao, L. Luo, L. Zhou, J. Zhang, B. Wang, *Biomed Res Int* **2019**, *2019*, 5756189.

[4] M. M. Salah, A. A. Ashour, T. M. Abdelghany, A. H. Abdel-Aziz, S. A. Salama, *Life Sci* **2019**, *239*, 116982.

[5] J. Wang, Y. Liu, H. Ding, X. Shi, H. Ren, *Stem cell research & therapy* **2021**, *12*, 15.

[6] M. Charni-Natan, I. Goldstein, *STAR Protoc* **2020**, *1*, 100086.

[7] H. Liu, J. Wang, Y. Ding, X. Shi, H. Ren, *Cell Death Dis* **2022**, *13*, 484.

[8] C. Zhang, R. An, Y. W. Bao, X. M. Meng, T. Q. Wang, H. N. Sun, F. M. Pan, C. Zhang, *Int Immunopharmacol* **2019**, *73*, 515.
